# Advances in electrical stimulation for wound healing

**DOI:** 10.3389/fbioe.2025.1662900

**Published:** 2025-11-19

**Authors:** Xiaofei Bi, Xiangchen Chen, Zheng Pang, Bihan Song, Hongxiang Li, Sicheng Feng, Hehe Jiang, Linlin Zhang, Haixiao Hu

**Affiliations:** 1 College of Traditional Chinese Medicine, Shandong University of Traditional Chinese Medicine, Jinan, China; 2 School of Pharmacy, Shandong University of Traditional Chinese Medicine, Jinan, China; 3 Innovative Institute of Chinese Medicine and Pharmacy, Shandong University of Traditional Chinese Medicine, Jinan, China; 4 College of Health, Shandong University of Traditional Chinese Medicine, Jinan, China; 5 College of Traditional Chinese Medicine, Beijing University of Chinese Medicine, Beijing, China; 6 Institute of Pharmacy (Institute of TCM Health Industrial Technology), Shandong University of Traditional Chinese Medicine, Jinan, China; 7 School of Materials Science and Engineering, Qilu University of Technology (Shandong Academy of Science), Jinan, China

**Keywords:** electrical stimulation, wound healing, cell proliferation, angiogenesis, therapeutic devices

## Abstract

Electrical stimulation (ES), as a cutting-edge biomedical strategy for promoting wound healing, accelerates tissue regeneration and repair processes through directional electric field intervention. Research demonstrates that both endogenous weak bioelectric potentials and exogenously applied electric fields can effectively guide cellular migration along electric field gradients while activating the biological activities of fibroblasts, keratinocytes, and endothelial cells. These mechanisms enhance collagen synthesis and accelerate angiogenesis, thereby significantly improving wound closure rates. This review comprehensively examines recent advancements in ES technology for wound healing, focusing on emerging applications of active microcurrent devices, passive microcurrent systems, and electroactive wound dressings. Particular emphasis is placed on innovative applications of conductive polymers (CPs) and nanocomposite materials in wound repair. By systematically analyzing the underlying mechanisms and therapeutic applications of ES in wound healing, this work aims to provide novel perspectives for optimizing ES technologies and facilitating their clinical translation, offering both theoretical significance and practical value in regenerative medicine.

## 1 Introduction

Human skin, a multilayered organ composed of the epidermis (a stratified squamous epithelium), dermis (connective tissue with vascular and neural networks), and subcutaneous tissue (adipose-rich hypodermis), serves as the primary defense system against external threats. This complex structure establishes a semipermeable barrier through diverse cellular populations and secretory products, effectively isolating microbial pathogens and environmental hazards. This system establishes a semipermeable barrier through diverse cellular populations and secretory products, effectively isolating microbial pathogens and other environmental hazards (Eming et al., 2014). However, wound formation occurs when this barrier integrity is compromised by surgical incisions, burn injuries, traumatic accidents, dermatological pathologies, microbial infections, or metabolic dysfunctions during tissue repair and regeneration processes.

Wound healing represents a complex biological process encompassing four sequential phases: hemostasis, inflammation, proliferation, and remodeling (Luo et al., 2021). During the hemostatic phase, vascular damage triggers rapid platelet aggregation at the injury site, forming a thrombus to prevent further blood loss. Concurrently, platelets release multiple growth factors (GFs) that initiate and promote subsequent inflammatory and proliferative phases (Scridon, 2022). The inflammatory phase involves leukocyte recruitment to the wound bed for necrotic tissue debridement, pathogen elimination, and foreign particle clearance, accompanied by inflammatory mediator secretion to prepare the healing microenvironment (García-Ramallo et al., 2002). In the proliferative phase, activated fibroblasts synthesize collagen and extracellular matrix (ECM) components, providing structural support for nascent tissue while promoting angiogenesis (ANG). This vascular network ensures oxygen/nutrient delivery and metabolic waste removal, essential for cellular viability and function (Smith and Rai, 2024). The remodeling phase, the final and prolonged stage, involves structural refinement of regenerated tissue. During this phase, excessive scar tissue undergoes gradual degradation through collagen fiber realignment and elastic fiber network reconstruction, ultimately restoring tissue architecture and functionality as close to pre-injury status as possible (Jang et al., 2023).

Acute wounds, typically resulting from surgical procedures, traumatic injuries, or radiation damage, demonstrate relatively short healing durations. In contrast, chronic wounds exhibit significantly prolonged healing cycles due to multiple interfering factors, including but not limited to diabetic foot ulcers and pressure ulcers associated with prolonged immobilization. These persistent wounds not only show delayed healing progression but also demonstrate heightened susceptibility to infection, ultimately imposing substantial patient discomfort and increased healthcare expenditures (Preetam et al., 2024).

ES emerges as a novel non-invasive/minimatically invasive therapeutic modality demonstrating potential in chronic wound management. Through elucidating ES’s biological mechanisms and optimizing treatment parameters, this approach offers innovative strategies for refractory wound care. ES facilitates healing through multimodal actions: modulating cellular behaviors, enhancing ANG, improving local microcirculation, and regulating inflammatory responses. Furthermore, ES upregulates GF expression and stimulates collagen synthesis, thereby accelerating tissue repair processes (Yang et al., 2022). Notably, emerging evidence suggests that ES functions synergistically with pharmacological agents (e.g., antimicrobials, growth factors) and bioactive materials (e.g., hydrogels, scaffolds), amplifying therapeutic outcomes through multimodal mechanisms (Qin et al., 2024).

The therapeutic concept of ES-mediated wound healing originated from early observations of bioelectrical phenomena, where endogenous weak currents were identified in biological systems. Subsequent advancements in bioelectrical signaling research have refined ES applications for tissue repair. Studies demonstrate ES accelerates healing through membrane potential modulation, enhanced cellular proliferation/migration, improved perfusion, and optimized wound microenvironments (Sundaram et al., 2021; Jin et al., 2018; Jiang et al., 2023). Recent mechanistic investigations have driven technological innovations, particularly in endogenous field regulation and exogenous delivery systems featuring microcurrent (MC) and pulsed current (PC) modalities (Wang et al., 2022). ES demonstrates dual clinical benefits: enhancing healing efficiency in chronic wounds while reducing infection risks, and improving patient quality of life with reduced healthcare burdens.

**FIGURE 1:**
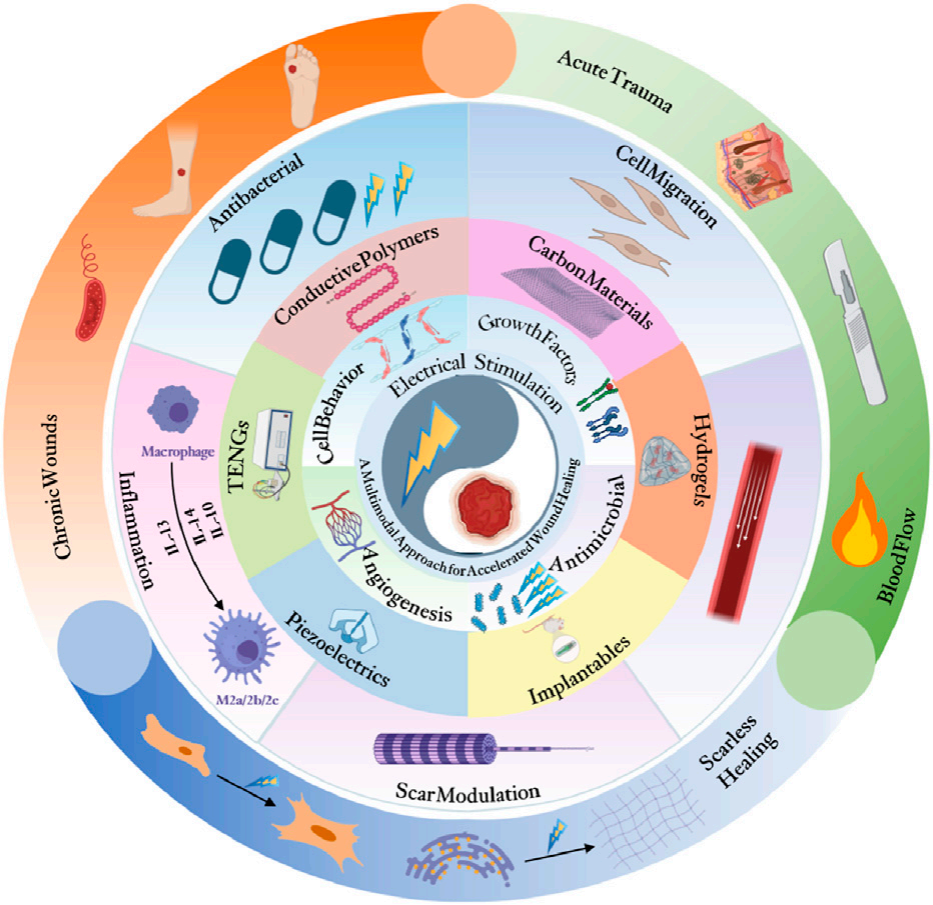
Electrical stimulation enhances wound healing through cellular activation and angiogenesis using advanced bioelectric technologies (Created with www.biorender.com).

As shown in Figure 1, this review comprehensively analyzes recent studies on ES-enhanced wound healing, systematically summarizing the latest therapeutic advancements. It examines the biological effects of various ES modalities (direct current [DC], PC, alternating current [AC]), evaluates material science breakthroughs including CPs and electroactive dressings (EDs), and identifies current challenges in ES-mediated healing to inform future research directions.

## 2 Principle

### 2.1 Classification of wounds and staging of wound healing

**FIGURE 2:**
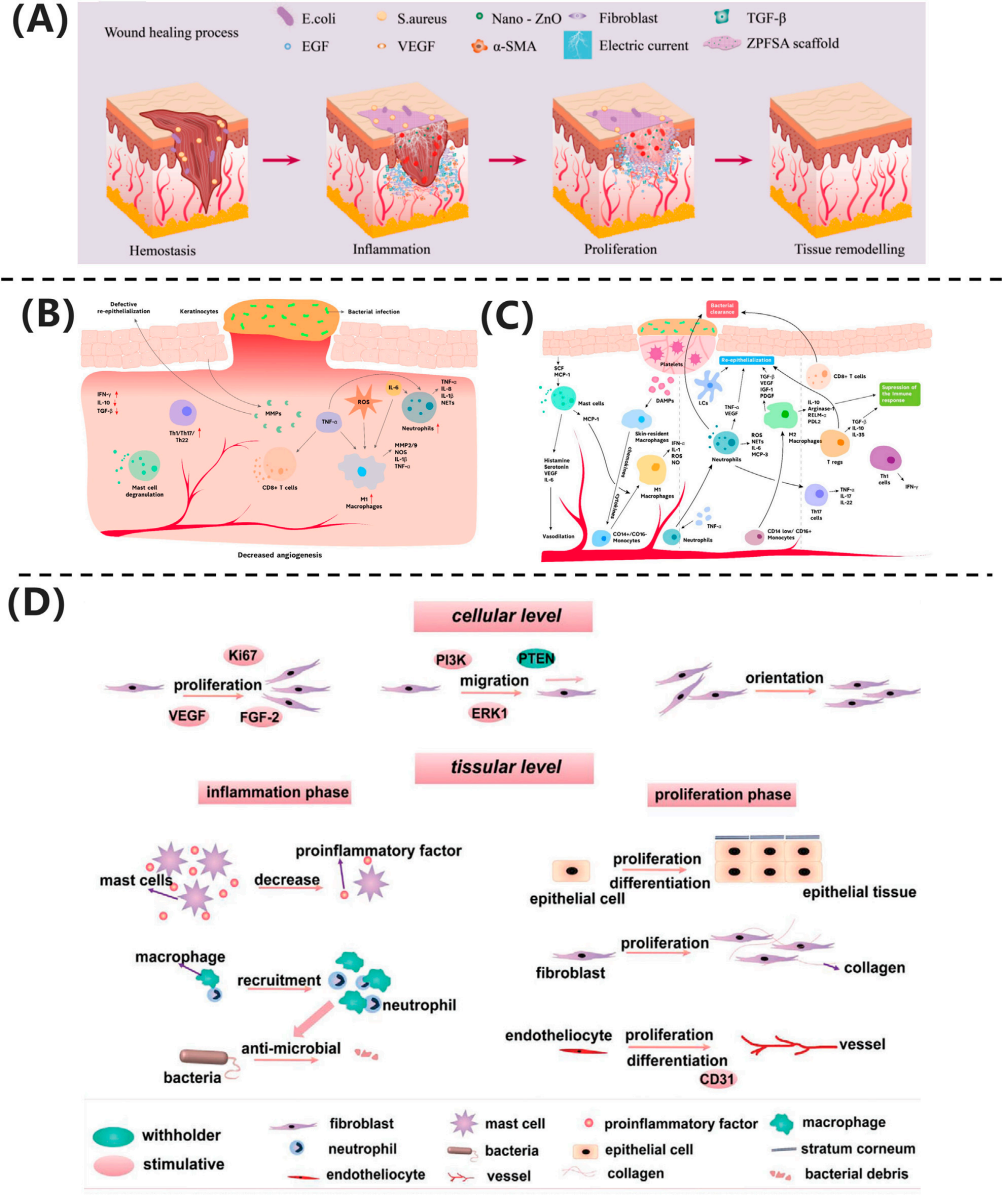
**(A)** ZPFSA scaffold promotes wound healing through piezoelectric microcurrents, enhancing cell migration and angiogenesis (Liang et al., 2022). Copyright 2022, ACS Publications. **(B)** Chronic wounds show persistent inflammation and impaired healing. **(C)** Acute wounds undergo coordinated immune response and tissue repair (Raziyeva et al., 2021). Copyright 2021, Biomolecules. **(D)** Electrical stimulation accelerates healing via cell proliferation and angiogenesis (Luo et al., 2021). Copyright 2021, Adv Healthc Mater.

Skin trauma refers to any injury that disrupts the natural anatomical structure of the skin, ranging from damage to the epidermal layer to deeper injuries involving subcutaneous tissues and organs. As shown in Figure 2A, skin wound healing progresses through four overlapping phases: hemostasis, inflammation, proliferation, and remodeling. The hemostasis phase primarily involves the formation of a “platelet plug” and activation of the “coagulation cascade” to achieve rapid physiological hemostasis. The inflammatory phase entails the activation of the immune system, with immune cells migrating to the wound site to clear necrotic tissue and create conditions for tissue regeneration. The proliferation phase is a critical stage of wound healing, where granulation tissue formation and epithelialization promote wound closure. Finally, during the remodeling phase, fibroblasts proliferate extensively, remodeling the wound with scar tissue to restore strength and further optimize its structure and function to approximate normal tissue.

From a biological perspective, wounds can be classified into acute and chronic wounds based on their healing speed and difficulty. As illustrated in Figure 2B, acute wounds heal through rapid and orderly immune regulation: neutrophils primarily clear pathogens, the dynamic polarization of M1/M2 macrophages balances inflammation and repair, and regulatory T cells (Tregs) suppress excessive immune responses. In contrast, chronic ulcers, depicted in Figure 2C, face healing obstacles due to a persistent inflammatory microenvironment. Mechanisms include aberrant M1 macrophage polarization, residual neutrophil extracellular traps (NETs), Th1/Th17-mediated cytokine storms, and microbial biofilm formation. Additionally, age-related reductions in repair factor secretion exacerbate extracellular matrix degradation and angiogenesis inhibition. The fundamental difference between these two types of wounds lies in the spatiotemporal dysregulation of cellular components and molecular mediators in their immune microenvironments (Raziyeva et al., 2021).

Liang et al. (2022) developed a dual-responsive regulatory model based on a ZnO/PVDF piezoelectric scaffold (ZPFSA), achieving phase-specific modulation: during the inflammatory phase, ZnO nanoparticles exert broad-spectrum antibacterial effects, inhibiting pathogen colonization and shortening the neutrophil-dominated inflammatory response; during the proliferation phase, piezoelectric microcurrents (ES) activate fibroblast migration and VEGF-mediated angiogenesis; and in the remodeling phase, transforming growth factor TGF-β signaling coordinates collagen fiber deposition while suppressing excessive α-smooth muscle actin (α-SMA) expression, enabling scar-free healing within 14 days.

Wound healing involves multiple cell types and is a complex, multi-layered, and coordinated process. Due to factors such as infection, traumatic scarring, systemic diseases, peripheral vascular diseases, and venous insufficiency, wounds often develop abnormal scars or progress into chronic wounds. Successful wound healing should restore normal bodily function while minimizing scar formation (Ibrahim et al., 2019). As shown in Figure 2D, electrical stimulation technology provides a molecular intervention strategy for pathological healing through phase-targeted regulation: during the inflammatory phase, it enhances macrophage chemotaxis by activating Kv channels, promotes neutrophil recruitment via ERK phosphorylation, and suppresses pro-inflammatory cytokines (TNF-α, IL-6) to accelerate inflammation resolution, while anode-mediated pH modulation synergistically inhibits bacterial colonization; during the proliferation phase, endogenous electric fields guide keratinocyte migration through PI3K/PTEN signaling, and microcurrents drive fibroblast proliferation and collagen synthesis via MAPK phosphorylation while upregulating VEGF to promote endothelial angiogenesis; in the remodeling phase, biphasic currents modulate TGF-β signaling to reduce abnormal collagen deposition and improve the biomechanical properties of scars. This multi-dimensional synergistic mechanism establishes a theoretical framework for phase-specific management of chronic wounds and scar regulation (Luo et al., 2021).

### 2.2 Changes in electrical signals in injured skin

In uninjured skin, there are potential differences between the cell membrane and the epithelial tissue layer, known as the “membrane potential” (∼70 mV) and the “trans-epithelial potential” (TEP, ∼10–60 mV), respectively. These potential differences are essential for the maintenance of cellular ion homeostasis. Negatively charged chloride ions at the surface of the skin and positively charged sodium ions in the dermis contribute to electrochemical processes that promote wound healing (Shapira et al., 2023). These potentials play an important role in maintaining tissue homeostasis and cellular function.

When the skin is injured, the balance of the transmembrane potential is disrupted, and the combination of cellular damage, ion channel opening, and inflammation after the injury leads to a decrease in the potential at the wound site, while the TEP around the wound remains unchanged, forming a low-resistance channel, resulting in a “short-circuitat the wound site”, and thus, the TEP drives the electric current flows out of the short-circuit site that occurs at the skin injury (Tai et al., 2009). used a vibration probe to measure the current at the human finger incision, and obtained the potential difference driving current.

**FIGURE 3:**
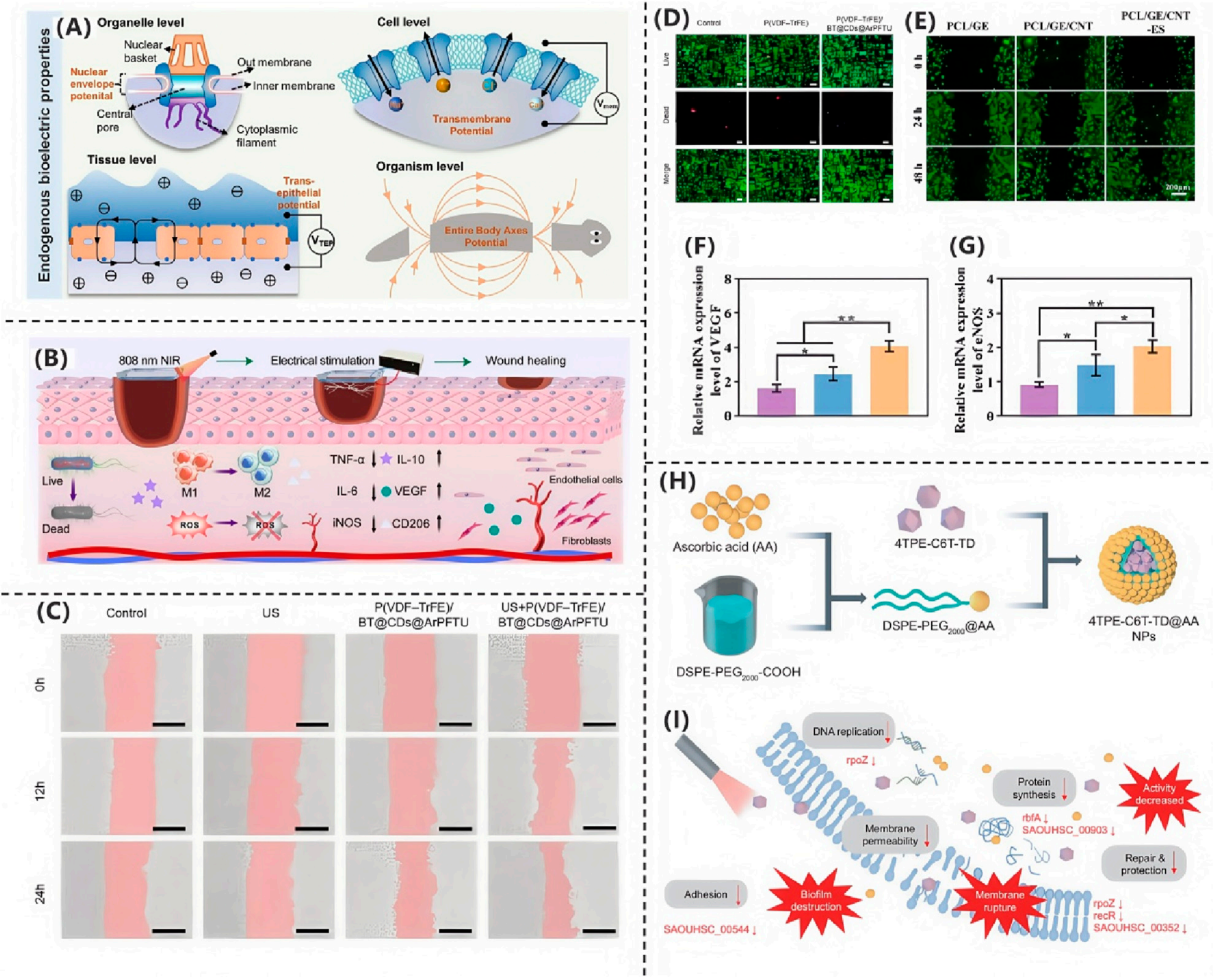
**(A)** Endogenous bioelectrical characteristics (Zhang Y. et al., 2025). Copyright 2025, Advanced Science. **(B)** PSMT hydrogel accelerates wound healing via multifunctional therapy (Zhang CK. et al., 2025). Copyright 2025, Chemical Engineering Journal. **(C)** L929 fibroblast migration under different treatments. **(D)** Fluorescence imaging of L929 fibroblasts (Shi et al., 2025). Copyright 2025, Nano Energy. **(E)** HUVEC scratch assay for migration. **(F,G)** VEGF and eNOS expression levels (Chen et al., 2025). Copyright 2025, Materials Today Bio. **(H)** Electrospun PVA/PCL scaffold with conductive modifications. **(I)** RNA-seq reveals bacterial metabolic disruption (Qian et al., 2025). Copyright 2024, Natl Sci Rev.

The research team led by Zhang Y. et al. (2025) systematically investigated the application mechanisms of electroactive electrospun nanofiber scaffolds in skin wound repair. This study comprehensively explored the biological effects of electric field-regulated tissue regeneration from a bioelectrical perspective. The findings demonstrate that bioelectrical signals, as fundamental characteristics of living organisms, are ubiquitous across various cell types. These signals manifest as endogenous electric fields (EFs), ionic currents, redox potentials, and transmembrane potential differences. During wound healing, such bioelectrical signals play a pivotal regulatory role. As illustrated in Figure 3A, when skin tissue is injured, the transepithelial potential (TEP) is disrupted, resulting in a significant potential gradient (∼200 mV mm^-1^) between the wound center and surrounding intact tissue. This lateral electric field directionally guides the electrotactic migration of epithelial cells and multiple repair-associated cells—including neutrophils, lymphocytes, monocytes, macrophages, endothelial cells, and fibroblasts—toward the wound center. Further analysis revealed that the ion dynamics of Cl^−^ and Na^+^ are critical determinants in maintaining wound potential. The concentration gradients of these ions not only regulate potential differences but also ensure the homeostasis of the wound’s electrical microenvironment through active transport via ion pumps. These findings confirm that endogenous electric fields serve as essential biophysical signals, not only orchestrating directional cell migration but also providing a theoretical foundation for novel wound management strategies based on electrical signal transduction.

### 2.3 Mechanisms of electrical stimulation for wound healing

In the study by Zhang CK. et al. (2025), the researchers developed a multifunctional composite hydrogel (PSMT) to promote infected wound healing through the combined application of near-infrared (NIR) photothermal therapy and ES. The hydrogel was fabricated by incorporating functionalized MXene@TA-Eu nanosheets (NSs) into a matrix composed of polyvinyl alcohol (PVA) and sericin. As demonstrated in Figure 3B, the PSMT hydrogel exhibited synergistic therapeutic effects under NIR-ES co-stimulation, including: Potent antibacterial activity, significantly reducing bacterial load; Modulation of the inflammatory microenvironment, suppressing pro-inflammatory cytokine release; Promotion of macrophage polarization toward the pro-healing M2 phenotype; Enhanced angiogenic capacity. These coordinated effects collectively accelerated the healing process of infected wounds.

#### 2.3.1 Influencing cell behavior

Normal skin electrical signals provide the basis for cells to maintain normal function, whereas changes in electrical signals in injured skin initiate cell migration mechanisms during wound healing, and bioelectricity increases the migration of key cells (e.g., fibroblasts, endothelial cells, etc.) to accelerate wound healing. Edwick et al. (2022) proposed that endogenous ES, with the aid of altered skin electrical signals, directs cellular movement along the direction of electric fields movement until wound closure and epidermal TEP is re-established (Korupalli et al., 2021). The absence of endogenous current reduces wound healing by approximately 25%, suggesting the value of endogenous ES associated with skin electrical signals in wound healing (Hampton and Collins, 2006; Rajendran et al., 2021). Exogenous EF, by applying an external current, is able to mimic endogenous currents, prompting the aggregation of key cells towards the centre of the wound, thereby accelerating wound closure. Electrical stimulation directly affects cell membrane potential, a process closely linked to skin electrical signals. Normal skin electrical signals maintain a steady state of cell membrane potential, and electrical stimulation further alters membrane potential after alteration of electrical signals in injured skin. When the EGFR is active, it activates multiple signalling pathways, including the MAPK-ERK1/2 and PI3K/Akt pathways. Preetam et al. (2024) pointed out that, in general, the MAPK-ERK1/2 are involved in multiple Phosphorylation of MEK contributes to cell migration and activates the downstream proteins ERK1 and ERK2. In addition, Hernández-Bule et al. (2014) have noted that static monophasic ES can control epithelial cell migration and proliferation by activating the ERK1/2 subunits of the MAPK signalling pathway.

The PI3K/Akt signalling pathway is critical for the cellular response to ES and has been extensively studied. ES significantly elevates the expression of the downstream protein PIP3, resulting in a heterogeneous distribution of Akt phosphorylation and consequently the distribution of cytoskeletal proteins towards the cathode. Meng Studies by have shown that the activation of the PI3K pathway is required for ES to drive NPC towards cathode-directed migration. Inhibition of PI3K/Akt by pharmacological or genetic means disrupts the electrophoretic phenomenon, highlighting its critical importance. In contrast, ES enhances Akt phosphorylation and PIP3 fluorescence, which also suggests that the PI3K/Akt pathway plays a key role in ES-induced NPC-directed migration (Meng et al., 2011). Arias-Calder et al. (2023) demonstrated that the proposed model of regulating FGF21 expression and secretion by electrical stimulation, which relies on extracellular ATP signalling and activation of the P2YR/PI3K/Akt/mTORC1 pathway in mouse skeletal muscle, whereas Syromiatnikova et al. (2024) demonstrated that basic fibroblast growth factor (FGF2 or bFGF) is essential for optimal wound healing. In addition, Zhao et al. (2006) demonstrated consistent external currents in human skin and rodent corneal and skin wounds, suggesting that phosphatidylinositol-3-OH kinase-g (PI(3)Kg) and phosphatases as well as tensin homologues (PTEN) control cellular orientated migration and identified the first gene that regulates cell motility and wound currents to promote wound healing.

In the study by Shi et al. (2025) researchers developed a flexible piezoelectric film, P(VDF-TrFE)/BT@CDs@ArPFTU, by incorporating functionalized barium titanate nanoparticles (BT@CDs@ArPFTU) into a poly(vinylidene fluoride-trifluoroethylene) (P(VDF-TrFE)) matrix via electrospinning. As shown in Figure 3C, cytocompatibility assays revealed that L929 fibroblasts cultured on the composite film exhibited comparable morphology and cell density to those in the control group and pure P(VDF-TrFE) group, confirming the material’s excellent biocompatibility. Further investigations demonstrated that under ultrasound (US) stimulation, the piezoelectric film not only maintained high biosafety but also significantly enhanced cell proliferation and migration. As shown in Figure 3D, quantitative analysis of cell migration via scratch assays showed that the US + P(VDF-TrFE)/BT@CDs@ArPFTU group achieved a 72.6% migration rate, outperforming both the control and US-only groups. These results indicate that the ultrasound-activated piezoelectric composite effectively potentiates cellular migration, highlighting its potential for applications in tissue regeneration and related fields.

#### 2.3.2 Regulation of growth factor expression

Both continuous DC stimulation and pulsed DC stimulation by modulating vascular endothelial growth factor (induce cell proliferation and achieve the promotion of wound healing the expression levels of) and related signalling factors, and subsequently modulating related signalling pathways. Sundaram et al. (2021) VEGF used a microfluidic bifurcation vascular model to study the endothelial permeability of DC stimulation. The study showed that 1 h of 70 v/m DC stimulation could induce vascular endothelial cell (VEC) proliferation by reducing the expression level of platelet endothelial cell adhesion molecule-1 (PECAM-1), activating VEGF receptor Inhibits B ithe phosphatidylinositol-3-kinase pathway and regulates vascular endothelial permeability through multiple signalling pathways to assist tissue regeneration and wound healing. By enhancing VEGF production by muscle cells, electrical stimulation induces significant angiogenesis *in vivo*, and Zhao et al. (2006) found that EFs as low as 75–100 mV·mm^-1^ (1.5–2.0 mV in endothelial cells) guided endothelial cell re-localisation, elongation, and migration in cultures.

These signalling pathways play a key role in regulating cell proliferation, migration and differentiation. Just as electrical signals from injured skin direct cells to migrate towards the centre of the wound, activated signalling pathways direct cells to carry out activities conducive to wound healing, such as accelerating division and proliferation to fill the wound vacancies, and guiding directional cell migration.

#### 2.3.3 Promoting angiogenesis

Angiogenesis refers to the formation of new blood vessels from the development of existing capillaries or post-capillary veins, which mainly includes: degradation of the vascular basement membrane during the activation phase; activation, proliferation, and migration of vascular endothelial cells; and reconstruction of the formation of new blood vessels and the vascular network, and it is a complex process involving multiple molecules from multiple cells.

Neovascularisation is a multifactorial, multistep evolutionary process, a variety of pathological conditions such as trauma healing, tissue regeneration and repair and tumour growth, metastasis are involved in vascular neovascularisation, which is also accompanied by changes in bioelectrical phenomena. VEGF has the physiological function of mediating angiogenesis. VEGF from a variety of cell types (including stromal cells) promotes budding through tip and stem cell formation, maintains tissue homeostasis during embryonic development and adult life, and is critical for embryonic and postnatal angiogenesis.

Exploring the PI3K/Akt pathway plays a key role in pro-angiogenesis. When the PI3K/Akt/mTOR pathway is activated, it transcriptionally activates the VEGF promoter, which increases the expression of VEGF. VEGF is an important protein that promotes the growth and remodelling of new blood vessels. VEGF is physiologically implicated in the regulation of angiogenesis and tissue repair, while pathologically associated with vascular anomalies in diverse conditions, including neoplastic disorders, ocular pathologies, chronic inflammatory diseases, and impaired wound healing. Notably, in the context of electrical stimulation-augmented wound repair, temporally regulated activation of the VEGF signaling pathway has been identified as a pivotal biological mechanism underlying the reconstruction of microvascular networks. Meanwhile, Wei et al. (2020) stimulated human umbilical vein endothelial cells (HUVECs) with EFs and assessed the activity and expression of eNOS. The PI3K/Akt-dependent pathway also activates eNOS by directly phosphorylating S1177, and the NO produced stimulates vasodilatation, remodelling, and angiogenesis. In addition, Akt signalling also increases poxia-inducible the protein level of hyfactor α. HIF-1α, in turn, further regulates the expression of downstream proteins involved in glucose metabolism and angiogenesis, such as vascular endothelial growth factor and erythropoietin, which further promote angiogenesis (Zhang et al., 2018). This pathway plays an integral role in a variety of physiological and pathological processes.

In the presence of fluid flow, electrical stimulation delivers a constant voltage accompanied by an electric current. The results suggest that the stimulation increases the permeability of the blood vessel-an important property that helps wound healing substances in the blood to reach the wound more efficiently.

The research team led by Chen et al. (2025) developed a conductive fibrous scaffold based on polycaprolactone/gelatin/carbon nanotube (PCL/GE/CNT), demonstrating that this electroactive wound dressing, when combined with exogenous ES therapy, significantly accelerates wound healing. Systematic RNA sequencing analysis elucidated the underlying molecular mechanisms, providing critical insights for skin tissue engineering applications. The combination of CNT composites and ES markedly enhanced both proliferation and migration of human umbilical vein endothelial cells (HUVECs), while upregulating angiogenesis-related genes. Scratch assays revealed that after 48 h of co-culture, the PCL/GE/CNT-ES group exhibited significantly higher HUVEC migration rates compared to control groups (PCL/GE and PCL/GE/CNT alone), as shown in Figure 3E, confirming the superior pro-angiogenic capacity of the combined therapy. Vascular endothelial growth factor (VEGF), a master regulator of angiogenesis during tissue repair, promotes wound healing by inducing neovascularization and granulation tissue formation. As shown in Figures 3F,G, VEGF receptor binding activates multiple pathways, including phosphorylated Akt (p-Akt), which orchestrates sustained endothelial cell functionality. Quantitative analysis of mRNA levels showed that the PCL/GE/CNT-ES group had significantly elevated expression of VEGF, eNOS, and p-Akt versus controls, suggesting the scaffold activates angiogenic signaling via VEGF upregulation. This study not only presents a multifunctional wound dressing but also deciphers its electro-bioactive mechanism, offering a translatable strategy for regenerative medicine.

#### 2.3.4 Enhancing antimicrobial defences

Electrophilic migration is a unique mechanism by which electrical stimulation promotes wound healing and is closely related to skin electrical signals. Hammerick et al. (2010) reported that mouse adipose-derived stromal cells (mASCs) migrated towards the cathode in physiological strength DC electric fields and showed dose-dependent migration. Zimolag et al. (2017) found that wound healing responded very rapidly to electric field stimulation, which occurred within 1 min. MSC migration towards the cathode and disruption of PI3K and Arp2/3 on had the most effect the electrophilicity of pronounced wound healing.

On the other hand, Guangping Tai pointed out that macrophages are another key participant in wound healing, migrating towards the anode, where external electric fields (related to the electrical signals of the injured skin) contribute to the migration by altering intracellular signals, activating small G proteins, and regulating the cytoskeleton, with fluctuating intracellular calcium ion concentrations involved in the regulation (Tai et al., 2018). It follows that electrophilicity further facilitates the coverage and connectivity of cells at the wound margin, enabling them to reach the wound site faster and clear pathogens (Buechler et al., 2021; Kindzelskii and Petty, 2000). Secondly, ES can inhibit biofilm formation by disrupting bacterial cell membrane structures or metabolic processes, increasing local metabolic activity and tissue oxidation, thus directly inhibiting bacterial growth and reproduction (Perez-Roa et al., 2006). In addition, ES contributes to the formation of a protective barrier on the wound surface, such as increasing collagen synthesis and deposition, further reducing the risk of infection.

In conclusion, wound healing is a complex and precise biological process involving multiple stages and coordinated interactions of different cells. Electrical signals play an important role in wound healing, and electrical stimulation, as a non-invasive therapeutic modality, accelerates the wound healing process by influencing cellular behavior, regulating growth factor expression, promoting angiogenesis, and facilitating the enhancement of antimicrobial defences through a variety of mechanisms. These findings not only reveal the intrinsic action principle of ES therapy, but also provide a solid theoretical basis for its wide application in clinical medicine (de Resende and Greene, 2008; Karavidas et al., 2006). Future studies should further explore the optimal stimulation parameters, therapeutic protocols, and safety issues of ES therapy to advance its in-depth development and wide application in clinical practice (Nagasaka et al., 2006).

In the study by Qian et al. (2025) the researchers designed and synthesized a near-infrared photothermal antibacterial agent, 4TPE-C6T-TD, as shown in Figure 3H. They then prepared 4TPE-C6T-TD@AA nanoparticles by encapsulating the agent in liposomes and modifying the surface with ascorbic acid (AA). Ascorbic acid not only served as a coating material but also enhanced the antibacterial performance of the nanoparticles due to its antioxidant and immunomodulatory functions. Experimental results demonstrated that under near-infrared light irradiation, these nanoparticles could partially inhibit biofilm formation and effectively eliminate preformed biofilms of *S. aureus* and *E. coli*. To systematically evaluate their antibacterial efficacy, the researchers employed colony-forming unit (CFU) assays and bacterial live/dead staining for quantitative analysis. The experimental data revealed that under 808 nm laser irradiation, 4TPE-C6T-TD@AA could achieve highly efficient clearance of *Staphylococcus aureus* and *Escherichia coli* (clearance rate >90%) by disrupting bacterial physiological functions, along with significant degradation of biofilms. Further mechanistic studies indicated that the antibacterial effect of these nanoparticles was closely related to interference with bacterial DNA expression processes. As shown in Figure 3I, transcriptomic analysis revealed that, compared to the control group, the differentially expressed genes in the 4TPE-C6T-TD@AA-treated group were significantly enriched in key pathways such as carbohydrate metabolism, bacterial infection and invasion, and DNA replication. These pathways play crucial roles in bacterial proliferation and virulence maintenance. Through a multi-target mechanism—combining physical photothermal killing and molecular-level metabolic disruption—4TPE-C6T-TD@AA synergistically inhibited bacterial proliferation and survival.

## 3 Electrical stimulation devices

### 3.1 Chemical micro-batteries

As shown in Table 1, power supply devices are primarily categorized into two types: external power sources and self-powered systems. In the field of wound healing, the application of electric field therapy has been limited by the bulky size of traditional external power sources and the output voltage instability of new power sources (Behr et al., 2021). In recent years, chemical micro-batteries have garnered attention due to their portability and cost-effectiveness (Zeng et al., 2022; Zhang et al., 2022). Sun XT. et al. (2024) reported a rechargeable flexible micro Zn-MnO2 battery (mZMB) that employs a ring-shaped circuit configuration to generate a ring-shaped electric field simulating the endogenous electric field (EEF) within the body. This electric field plays a crucial role in regulating cellular activities, thereby accelerating the healing process.

**TABLE 1:** The summary of skin wound healing by electrical stimulation of traditional power supply.

Device	Type of current	Indensity/duration/frequency of electrical stimulation	Animal of model	Effect of wound healing	Ref.
Chemical microcell	Direct current	Lattice pattern with an electrode spacing of 2 mm and a maximum electric field strength of 328.5 V/m	Diabetic C57BL/6 mice (STZ-induced, blood glucose level > 300 mg/dL, 6-mm full-thickness skin wounds)	96.4% healing rate at 24 days (32.7% improvement); 2.1-fold increase in CD31^+^ expression; 64.8% reduction in epithelial gap.	Kim et al. (2024c)
TEN	Drug release: Biphasic current (±3 μA) with a voltage of ±20 V and a frequency of 2 Hz; Cell stimulation: 5 μA direct current (DC)	Duration: 30 min per session Frequency: Twice or thrice daily (with 30-min intervals)	Hu02 human fibroblasts	54% higher cell proliferation (154% viability); 40% enhanced scratch closure rate; 100% cumulative drug release (37 μg/mL within 2 h).	Nazari-Vanani et al. (2024)
	Pulse current	Output voltage range: 44.87–69.51 mV (average range: 54.11–60.87 mV)	Full-thickness skin defect model in diabetic mice with 8 mm diameter wounds	86.3% healing rate at 7 days (219% acceleration); 93.9% collagen deposition area (+78% vs. control); 374.7/mm^2^ microvascular density (+499% vs. control).	Zhang et al. (2024d)
	Low-intensity alternating current (AC) electric field with a voltage range of ±1 V to ±8 V	Peak power density: ∼30 mW/m^2^ (under a 200 MΩ load and 50 N pressure) Current range: 5–40 nA (animal studies)	Full-thickness skin defect model in *Staphylococcus* aureus-infected mice (dorsal wound diameter: 8 mm)	22.7% healing rate at day 5; complete healing by day 10; 4.5-fold increase in fibroblast activity (±8 V stimulation).	Du et al. (2021)
Implant system	Piezoelectric dc pulse	Output voltage: Peak voltage of 3.23 V under a load of 1 N. Magnetoelectric coefficient: 18.97 mV cm^-1^ Oe^-1^ at a frequency of 1,400 Hz.	Full-thickness skin defect model in rats with 10-mm-diameter circular wounds	94.6% healing rate at day 4; 1.88-fold higher collagen content; 9.3-fold increase in CD31^+^ expression (day 7).	Zhang et al. (2024a)
Piezoelectric generator	Alternating current (AC) pulses induced via mechanical stress, with an output voltage of 35 V at 4% strain and 18 V under 50 N pressure	*In vitro* experiments: Parameters included 0.5 Hz frequency, 4% strain amplitude, administered three times daily (30 min per session). *In vivo* experiments: Parameters included 50 N pressure, 5 Hz frequency, applied twice daily (10 min per session).	3-month-old SD rats with full-thickness dorsal skin defects (3 mm × 8 mm)	61.5% wound area reduction at day 4 (23% acceleration); 19.9% reduction at day 7 (59% acceleration); 76% elevation in collagen type I expression.	Xu et al. (2024a)
Self-powered equipment	AC	Output parameters: Frequency 1–4 Hz, Voltage 70–130 V, Current 2–6 μA	Male BALB/c mice with full-thickness *Staphylococcus aureus* (*S. aureus*)-infected wounds in the dorsal region	4.4% residual wound area at day 12 (73.3% reduction); 0.2% bacterial survival rate (99.8% inhibition); 2.3-fold higher collagen density.	Zhang et al. (2024b)

Godwinraj and George (2021) developed a wireless closed-loop smart bandage with low impedance and adjustable adhesion through dual-conductive hydrogel electrodes. This bandage has demonstrated significant efficacy in accelerating wound healing speed and enhancing skin remodeling, with improvements of approximately 25% and 50%, respectively. Experimental studies demonstrate that electrical stimulation significantly accelerates wound closure and induces a marked elevation in wound impedance, facilitating rapid attainment of impedance plateau. This integrated approach enables concurrent real-time monitoring and active therapeutic modulation of wound healing processes.

### 3.2 Triboelectric nanogenerators (TENGs)

Self-powered systems have become a research hotspot due to their portability, high safety, and cost-effectiveness. TENGs as a class of innovative energy harvesting devices, have emerged as a preferred solution for numerous electronic applications owing to their excellent flexibility and optimized performance (Cheah et al., 2021). TENGs operate based on the principle of contact electrification, where the contact and subsequent separation of two dissimilar materials induce charge redistribution, generating alternating current (AC) (Long et al., 2018). The independent triboelectric layer design allows TENGs to function without the need for moving parts or direct circuit connections, significantly improving efficiency and enabling non-contact operation. Depending on the materials used, TENGs can be categorized into solid-solid, solid-liquid, and liquid-liquid types, where the effective contact area directly influences the current output.

**FIGURE 4:**
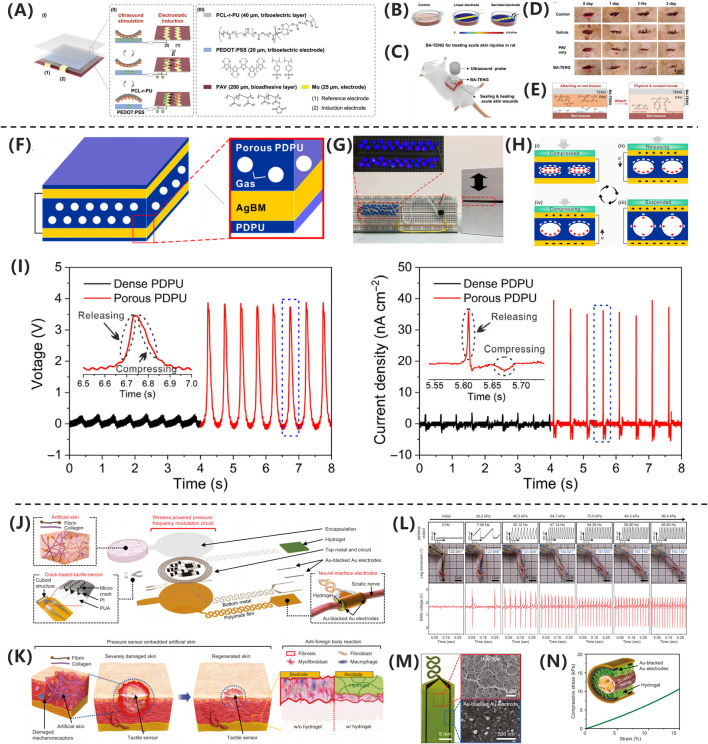
**(A)** BA-TENG generates electric field for wound healing. **(B)** Sawtooth electrodes enhance field intensity. **(C)** BA-TENG promotes healing via adhesion and stimulation. **(D)** BA-TENG enables chemostasis and cell migration. **(E)** PAV adhesive bonds to wet tissue (Meng et al., 2023) Copyright 2023, Adv Material. **(F)** GS-TENG design for power generation. **(G)** GS-TENG powers LEDs. **(H)** GS-TENG working mechanism. **(I)** Porous PDPU enhancements output (Kang et al., 2024). Copyright 2020, Sci. Adv. **(J)** WTSA components and design. **(K)** CFAS enhancements skin regeneration. **(L)** Gradient pressure correlations with motion. **(M)** Hydrogel coated electrodes improve biocompatibility. **(N)** Gelation hydrogel matches nerve mechanisms (Xiong et al., 2020). Copyright 2024, Nat Commun.

Meng et al. (2023) developed a novel bioadhesive TENG(BA- TENG) made from novel biocompatible polymer, as shown in Figure 4A, aiming to achieve rapid closure of acute wounds and accelerated healing through electrical stimulation. As shown in Figures 4B-E, the BA-TENG consists of a flexible and biocompatible TENG as the upper layer and a bioadhesive layer as the lower layer, enabling strong adhesion on wet tissues. Under ultrasonic actuation, the device generates a stable electric field (approximately 0.86 kV·m^-1^), which accelerates wound healing by promoting cell migration and proliferation. Experimental results demonstrate that the BA-TENG achieves immediate wound closure in an *ex vivo* porcine colon model within approximately 5 s and significantly reduces blood loss (by about 82%) and hemostasis time in an *in vivo* rat model. This device showcases potential for application in emergency scenarios, providing new insights and methods for electrical stimulation-enhanced wound healing.

Wang WJ. et al. (2024) developed a novel aqueous-aqueous triboelectric nanogenerator (A-A TENGs)-powered multifunctional wound healing system by combining polyethylene glycol and dextran solutions, creating a 100% contact interface to enhance charge transfer and improve current output. The A-A TENGs generate charges through the contact and separation of two aqueous materials, forming a current through an external circuit. As integrating functionalized conductive hydrogels, the system ensures uniform distribution of the electric field at the wound site, promoting fibroblast migration and proliferation, enhancing angiogenesis, increasing collagen deposition, eliminating bacteria, and reducing inflammatory cells. This significantly accelerates the wound healing speed in infected wounds, demonstrating its potential for electrical stimulation-enhanced wound healing.

In the study by Xiong et al. (2020) as shown in Figure 4F, the researchers developed a gas-solid interactive triboelectric nanogenerator (GS-TENG) based on a viscoelastic porous polydimethylsiloxane-dimethylethylenedioxime polyurethane (PDPU) elastomer. The elastomer, with a thickness of 3 mm and an average pore size of 0.6 mm, features uniformly distributed closed pores that enable efficient gas trapping. A highly transparent and conductive silver nanowire bundle mesh (AgBM) was employed as the electrode material. Through a precision transfer process, the AgBM was tightly integrated with the porous PDPU substrate and subsequently coated with a 0.5 mm-thick dense PDPU protective layer. This structural design ensures excellent conductivity while maintaining 70% optical transmittance. The working mechanism of the GS-TENG relies on periodic gas-elastomer interfacial interactions, which couple triboelectrification, electrostatic induction, and dynamic charge migration in the gas phase. As shown in Figure 4G, this synergistic effect enables instantaneous illumination of dozens of serially connected commercial LEDs. The operational process can be divided into four distinct stages, as shown in Figure 4H: (i) Under external pressure, the PDPU undergoes compressive deformation, enhancing contact between pore surfaces and facilitating charge transfer from the gas to the negatively charged PDPU, forming an electric double layer with a negatively charged surface and positively charged gas; (ii) Upon pressure release, the positively charged gas undergoes potential redistribution via dynamic charge migration. Due to pore structure heterogeneity, transient asymmetric charge distribution occurs, driving free electrons from the top electrode to the bottom electrode through electrostatic induction, generating a forward current; (iii) At full unloading, the system reaches potential equilibrium, resulting in zero output signal; (iv) When pressure is reapplied, reverse charge transfer induces electron backflow, producing a negative current. Continuous alternating current output can thus be achieved under periodic mechanical excitation. As shown in Figure 4I, comparative experiments demonstrate that conventional TENGs using dense PDPU structures only yield weak outputs of 0.2 V, 2.5 nC, and 1.2 nA cm^−2^ in terms of voltage, charge quantity, and current density, respectively.

### 3.3 Implantable power devices

Huang et al. (2024) demonstrated a body fluid (BF)-activated metal-based implantable battery, an ideal self-powered device for wound therapy. They developed a tubular Mg-Mo battery to promote wound healing. By evaluating electrical stimulation under body fluid conditions, the study correlated discharge current, dissolved oxygen (DO) concentration, and serum organics simulated with fetal bovine serum (FBS). Effective stimulation persisted for over 5 days, with a current density of 25–400 μA·cm^-2^ in phosphate-buffered saline. The *in vivo* DO concentration supported normal battery discharge. Although the addition of FBS reduced the voltage (while still meeting effective stimulation levels), it extended the discharge duration. Additionally, a body fluid-driven battery was implanted between the hindlimb muscles to deliver ES, accelerating full-thickness skin wound healing in rats. Compared to the blank control group (75.11% ± 0.41% healing rate at 14 days), rats receiving battery stimulation for 5 days (BS-5 days) exhibited a significantly improved healing rate of 97.50% ± 0.29%. This work proposes an innovative therapeutic strategy for wound healing with potential clinical applications.

Baniya et al. (2023) developed a wearable bioelectronic system composed of a polydimethylsiloxane (PDMS) device and a printed circuit board (PCB). The system features a modular design that continuously drives H^+^ ions through a cation-selective hydrogel-filled capillaries into the wound bed by applying a positive voltage between the working electrode (WE) and reference electrode (RE). This process replaces endogenous sodium ions, reducing the M1/M2 macrophage ratio by 35.86% in a mouse model, demonstrating the system’s efficacy in enhancing wound healing. Kang et al. (2024) developed a fully implantable wireless powered tact sensor system embedded artificial skin (WTSA) that integrates biologically artificial skin and crack based tact sensors, as shown in Figures 4J,K. The system employs a hydrogel coating to reduce foreign body reactions, thereby enhancing the effectiveness of electrical stimulation. The crack-based tactile sensor detects externally applied pressure, converting it into frequency-modulated electrical signals. As shown in Figures 4L-N, these signals stimulate the scientific nerve to induce corresponding muscle responses, effective triggering leg movement. This mechanism promotes the regeneration of the intact skin layer, demonstrating excellent biocompatibility and healing-promoting potential. This work provides novel insights and an empirical foundation for electrical stimulation applications in wound healing. Li et al. (2024) proposed a wearable therapeutic zinc battery that utilizes poly (3,4-ethylenedioxythiophene) (PEDOT) -based polyelectrolyte hydrogel as epidermal stimulation electrodes to eliminate wound infection and promote diabetic wound healing. The battery consists of a zinc anode and a PEDOT-based conductive polymer hydrogel cathode. PEDOT, a cationic polymer, interacts with the negative charges on bacterial cell membranes, disrupting their integrity. The redox reactions of PEDOT generate ROS, such as H2O2, which damage critical bacterial organelles, including DNA, RNA, and proteins. Additionally, PEDOT + reacts with glutathione (GSH), depleting GSH in bacterial cells and reducing their ability to scavenge ROS, thereby enhancing its bactericidal effect. The anode leverages the redox reaction of zinc to produce zinc ions with antibacterial properties, which inhibit bacterial biofilm formation and promote DNA replication, transcription, and cellular damage repair.

The microcurrent generated by the battery mimics the endogenous electric field of the body, promoting fibroblast migration and angiogenesis, while reducing the expression of inflammatory factors. Zinc ions further enhance cell proliferation and migration, and activate the MAPK and PI3K/Akt signaling pathways. The PEDOT-based conductive polymer hydrogel exhibits excellent biocompatibility and mechanical properties, providing an optimal growth environment for cells and facilitating cell-cell interactions, thereby accelerating wound healing.

### 3.4 Implantable systems

In recent years, technological advancements have driven the development of specialized therapeutic approaches that extend beyond direct electrical stimulation of wounds. These innovative methods indirectly regulate wound healing by modulating various factors.

Sun YN. et al. (2024) developed a self-powered wound dressing using polyvinylidene fluoride (PVDF) as the substrate, loaded with vancomycin (VAN) and carboxylated carbon nanotubes (c-MWCNTs). This dressing combines a Lock-ON/OFF electric field-driven drug release mechanism with electrical stimulation therapy to accelerate the healing of infected wounds. The dressing was fabricated using electrospinning and soft template methods, maintaining a closed state for drug release under non-stimulated conditions. When mechanical stress is applied, an electric field is generated through the piezoelectric effect, enabling precise drug release under stimulated conditions. The cumulative drug release rate reached 88.57%, representing an 89-fold increase compared to the non-stressed group. Through electrical stimulation, the device effectively promoted wound healing, achieving a healing rate of 100% within 10 days, demonstrating excellent antibacterial efficacy and the ability to accelerate tissue regeneration. Wang H. et al. (2024) investigated a capacitive antibacterial dressing composed of a polypyrrole-wrapped carbon cloth and a bacterial cellulose (BC) hydrogel separator. The device demonstrated a sterilization efficiency of up to 99.97% after applying a 1 V voltage for 10 min, with a kill rate of 99.99% against multidrug-resistant bacteria. Electrical stimulation not only enhanced the antibacterial capability of the dressing but also significantly accelerated wound healing in a mouse model of infected full-thickness skin defects by promoting local collagen deposition and microvascular reconstruction, indicating the promising potential of this capacitive antibacterial dressing in promoting wound healing.

**FIGURE 5:**
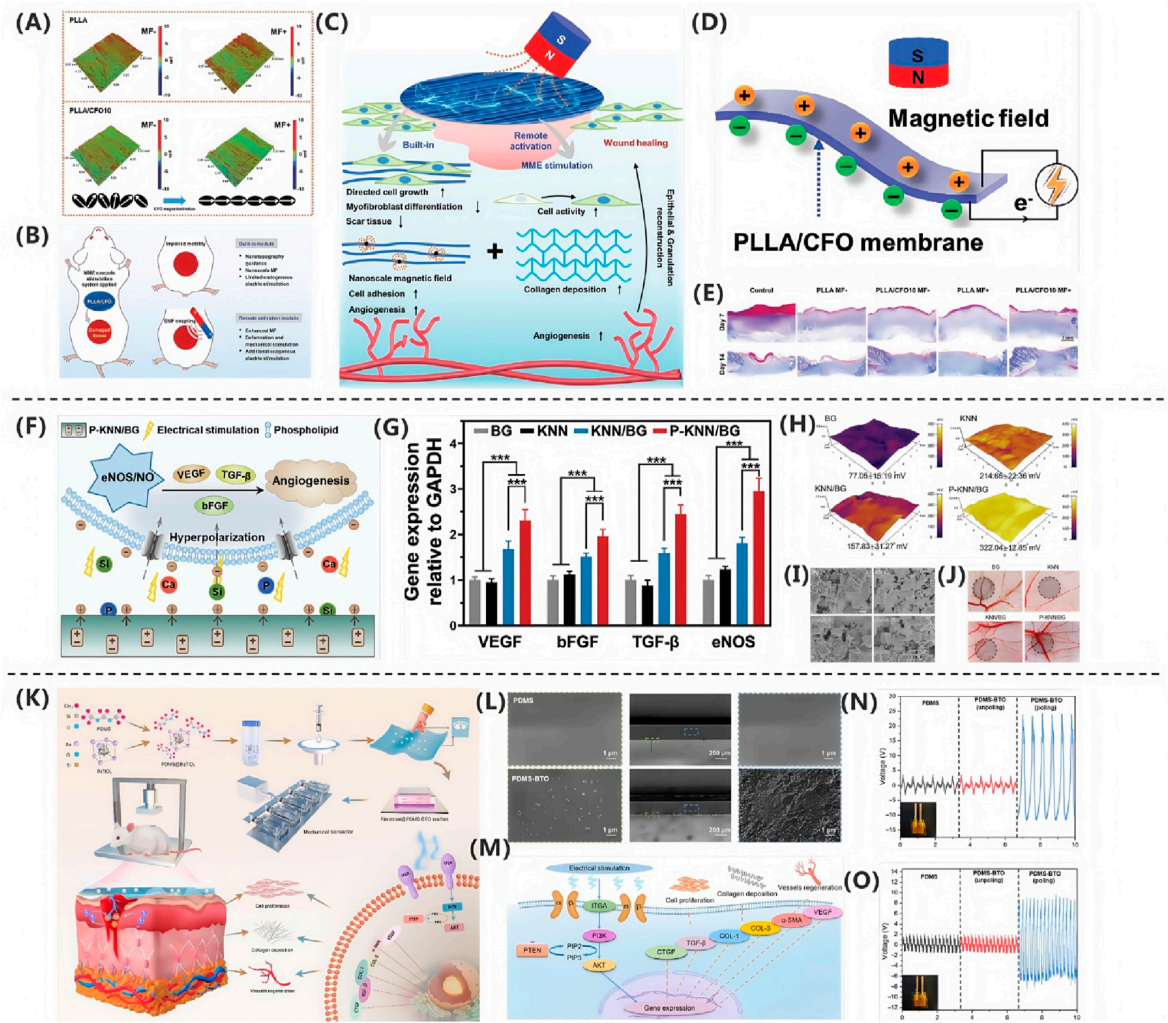
**(A)** PLLA/CFO10 membranes showed reduced roughness under EMF. **(B)** EMF-triggered CFO realignment activated PLLA piezoelectricity. **(C)** PLLA/CFO10 enhanced collagen alignment post-op. **(D)** P-KNN/BG promoted angiogenesis via Ca^2+^/Si/P signaling. **(E)** P-KNN/BG upregulated pro-angiogenic genes (Zhang Q. et al. (2024)). Copyright 2024, Adv Funct Mater. **(F)** SKPM revealed high surface potential in P-KNN/BG. **(G)** FE-SEM confirmed uniform morphology. **(H)** CAM assays showed P-KNN/BG enhanced vascularization. **(I)** BaTiO_3_/PDMS activated PI3K/AKT pathway. **(J)** SEM verified BaTiO_3_ dispersion in PDMS (Li CH. et al. (2023)). Copyright 2023, Adv Healthc Mater. **(K)** Piezoelectric signals upregulated ECM/VEGF via integrin-PI3K. **(L)** Polarized PDMS-BTO generated 35 V. **(M)** PDMS-BTO produced higher voltage under impact. **(N)** Porous PDPU achieved higher charge density. **(O)** PDMS-BTO outperformed pure PDMS (Xu Q. et al., 2024). Copyright 2024, Nano Res.

In the study by Zhang Q. et al. (2024), an innovative magneto-mechano-electric (MME) cascade stimulation system for wound healing was developed and validated. As shown in Figure 5A, mechanistic studies revealed that pure PLLA membranes maintained stable surface morphology and roughness parameters (Ra = 1.47 μm, Rq = 1.84 μm, Rt = 19.29 μm) before and after magnetic field stimulation, while PLLA/CFO10 composite membranes exhibited significant reduction in surface roughness (Ra = 1.05 μm, Rq = 1.34 μm, Rt = 17.18 μm) under magnetic field exposure. This system, as illustrated in Figure 5B, employs aligned poly (L-lactic acid)/cobalt ferrite (PLLA/CFO) magnetoelectric nanofiber membranes as the core component, working synergistically with a remotely controllable magnetic field to achieve therapeutic effects. This deformation effect originates from the local mechanical strain induced by the aligned magnetic dipole arrangement of CFO nanoparticles, which is subsequently transferred to the piezoelectric PLLA matrix through interfacial coupling, ultimately leading to surface polarization changes and electrical signal output, as shown in Figure 5C. Regarding material selection, PLLA, as an FDA-approved implantable biomaterial, exhibits excellent biocompatibility, biodegradability, structural stability, and piezoelectric properties; meanwhile, polydopamine (PDA)-modified CFO nanoparticles not only demonstrate outstanding magnetic characteristics but also significantly enhance interfacial bonding strength and biostability with the PLLA matrix. As shown in Figure 5D, the composite membrane can generate magnetic field-driven electrical stimulation output, with its magnetoelectric conversion performance systematically verified. Through systematic *in vitro* cellular experiments and full-thickness skin defect models in rats, the MME system demonstrated remarkable therapeutic advantages: promoting cell adhesion, proliferation and spreading behavior, accelerating wound healing processes, and enhancing collagen deposition and angiogenesis. Notably, as shown in Figure 5E, quantitative analysis via Masson’s trichrome staining revealed that the magnetic field-treated PLLA/CFO10 experimental group exhibited the most significant collagen deposition, confirming its exceptional performance in tissue regeneration.

### 3.5 Multi-layer stacked electrets

Kim SW. et al. (2024) proposed a novel multi-layer stacked electret (MS-electret) patch, which is fabricated using a corona charging system and is capable of self-generating a sustained direct current electric field (DCEF) without an external power source. The design of the MS-electret allows for the modulation of electric field strength by physically stacking dielectric layers, providing stable electrical stimulation. This significantly inhibits the transformation of human dermal fibroblasts (hDFs) into myofibroblasts, reducing fibrotic activity. In the 7-layer MS-electret treatment group, the expression of the COL1A1 gene was significantly reduced by approximately 90%, while the expression of the COL3A1 gene increased, indicating the device’s remarkable efficacy in promoting wound healing and inhibiting scar formation.

Compared to other materials, the OPV cell can generate electricity continuously without the need for battery replacement or connection to a power line. Both the OPV cell and MEMS electrode exhibit excellent flexibility, allowing them to adapt to the movement of the skin surface. The dressing also demonstrates good biocompatibility and antibacterial performance, promoting wound healing and reducing healing time. *In vivo* experiments and proteomic analysis revealed that the proposed PMH dressing significantly accelerates the healing of infected diabetic wounds by enhancing extracellular matrix regeneration, eliminating bacteria, modulating inflammatory responses, and regulating vascular function. Therefore, the PMH dressing represents a powerful, versatile, and effective solution for diabetic wound care, paving the way for the development of electrical stimulation wound dressings.

Qiu et al. (2020) fabricated a multilayer composite membrane (MC membrane) using electrospinning technology, composed of an antibacterial layer (ABL), a healing-promoting layer (HPL), and a reinforcement layer (RFL) made from zein/ethyl cellulose (zein/EC). By utilizing protoporphyrin (PPIX) as a photosensitizer, the membrane achieved photodynamic antibacterial effects. Additionally, electrical stimulation was employed to promote cell migration and angiogenesis, while reducing inflammatory responses. This approach significantly enhanced the wound healing rate in a mouse skin defect model, achieving a healing rate of 92.4% by the 10th day.

### 3.6 Piezoelectric generators

Zhang et al. (2023) developed a piezoelectric generator (PEG) based on poly (L-lactic acid) (PLLA) and vitamin B2 (VB2). Compared to traditional generators, the PLLA/VB2 PEG is made from bio-based materials, offering superior biocompatibility and greater suitability for skin wound treatment. By blending PLLA and VB2, they improved the crystal structure and β-phase orientation, resulting in better biocompatibility and higher piezoelectric output than commonly used polyvinylidene fluoride (PVDF)-based PEGs. The PLLA/VB2 PEG also boasts advantages such as non-invasiveness, biodegradability, and low cost, making it highly promising for future applications.

Liang et al. (2022) utilized 3D printing technology to fabricate a novel ZnO nanoparticles modified PVDF/sodium alginate (SA) piezoelectric hydrogel scaffold (ZPFSA), providing a new therapeutic option for rapid wound healing and scar prevention. The scaffold features a dual piezoelectric release mode in both vertical and horizontal directions, ensuring continuous generation of piezoelectric current. As a bioelectric stimulation signal, it triggers a series of responses, including cell proliferation and migration, ordered collagen deposition, neovascularization, and upregulation of various growth factors related to wound healing. This ultimately achieves rapid and complete wound healing in a rat full-thickness wound model. The material’s swelling properties enable current generation in the vertical direction, making it essential to test its swelling performance. When the concentrations of sodium alginate and PVDF are optimized for printing, the piezoelectric scaffold achieves a swelling rate of approximately 331%, establishing a swelling-based vertical piezoelectric response mode. The material also exhibits high mechanical performance, ensuring stable current generation under high-frequency motion on the skin surface.

Yu et al. (2024) developed a single-electrode TENG skin patch using molybdenum disulfide (MoS2) and biocompatible gelatin methacryloyl (GelMA) hydrogel. MoS2 exhibits excellent electrical conductivity and photothermal conversion properties, while GelMA demonstrates outstanding biocompatibility. The TENG device is capable of harvesting biophysical energy, generating an electric field around damaged tissues, and enhancing wound healing through near-infrared (NIR) photothermal effects. Additionally, the TENG can function as a real-time sensor to monitor physiological signals. The TENG prototype achieved a peak voltage output of 48.80 V and a current output of 0.57 μA.

*In vitro* experiments using mouse fibroblastsdemonstrated that the TENG accelerates cell migration through the combined effects of photothermal heating and real-time electrical stimulation. Animal studies further confirmed that the TENG effectively promotes collagen deposition and angiogenesis, ultimately enhancing tissue regeneration and wound healing. Notably, this study is the first to report the use of a MoS2-based TENG for accelerating wound healing. This work is expected to not only provide new avenues for the application of self-powered wearable electronics in wound therapy but also highlight their potential in advanced sensing systems.

The research team led by Li CH. et al. (2023) successfully developed a polarized potassium sodium niobate (K_0_._5_Na_0_._5_NbO_3_)-based piezoelectric bioactive glass composite (P-KNN/BG). This innovative material combines a bioactive glass (BG) matrix with lead-free piezoelectric KNN through polarization treatment, integrating BG’s exceptional osteointegration capacity with KNN’s piezoelectric properties. As shown in Figure 5I, field-emission scanning electron microscopy (FE-SEM) analysis revealed uniformly distributed surface morphology across all experimental groups. Through incorporation of KNN piezoelectric components and optimized polarization processing, the composite achieved a significantly enhanced surface potential of 322.04 ± 12.85 mV, representing a 3.2-fold increase compared to pure BG (77.05 ± 15.19 mV),as shown in Figure 5H. This potential enhancement stems from two synergistic mechanisms: (1) KNN doping imparts tunable dielectric properties, and (2) polarization treatment markedly improves piezoelectric performance via domain alignment. Notably, piezoelectric effect-induced electrostatic interactions promote accelerated release of bioactive ions (Ca^2+^, Si^4+^, and PO_4_^3-^) from the BG matrix. At optimal concentrations, these ions effectively stimulate secretion of angiogenesis-related factors. The molecular mechanism, illustrated in Figure 5F, demonstrates P-KNN/BG’s dual-mode pro-angiogenic action: Piezoelectricity-induced membrane hyperpolarization establishes electrochemical gradients facilitating ion influx. Sustained ion release activates the eNOS/NO signaling pathway, significantly upregulating expression of angiogenic factors (VEGF, b-FGF, and TGF-β), as shown in Figure 5G. As shown in Figure 5J, *in vivo* chick chorioallantoic membrane assays confirmed that P-KNN/BG treatment not only exhibited excellent biocompatibility but also increased vascular network density by 2.3-fold while promoting extensive neovascularization. This study provides novel design principles and therapeutic strategies for developing electrically active bone repair materials.

Qi Xu’s research team (Xu Q. et al., 2024) successfully developed a self-powered repetitive mechanical impact-electrical stimulation (RMI-ES) system based on a barium titanate/polydimethylsiloxane (BaTiO_3_/PDMS) piezoelectric composite membrane, as shown in Figure 5K. The core component of this system consists of non-centrosymmetric tetragonal-phase BaTiO_3_ (T-BTO) nanoparticles uniformly dispersed in a PDMS matrix, which, after polarization treatment, forms a flexible composite membrane with piezoelectric properties. Scanning electron microscopy (SEM) analysis revealed that the T-BTO nanoparticles were homogeneously distributed within the PDMS matrix, a structural design that endows the material with both the excellent biocompatibility and flexibility of PDMS and the highly efficient electromechanical conversion capability of T-BTO, as shown in Figure 5L. Under mechanical stimulation, the composite membrane demonstrated remarkable energy conversion performance, generating an output voltage of approximately 35 V at 2 Hz frequency and 4% strain, as shown in Figure 5N, and 18 V under 5 Hz frequency and 50 N impact loading, as shown in Figure 5O. This mechanoelectrical energy conversion arises from the non-centrosymmetric lattice structure of T-BTO, which produces microscopic piezoelectric polarization under external force, thereby generating a dynamic built-in electric field. As shown in Figure 5M, mechanistic studies demonstrated that the electrical stimulation generated by the piezoelectric membrane exerts therapeutic effects by activating the PI3K/AKT signaling pathway: the electric field promotes PI3K-catalyzed generation of PIP_3_, which subsequently activates AKT serine/threonine kinase, triggering a cascade of intracellular events that regulate critical biological processes, including cell survival, proliferation, and migration. Additionally, this pathway participates in modulating angiogenesis and extracellular matrix remodeling. Systematic *in vitro* and *in vivo* animal model experiments confirmed the system’s significant pro-healing effects, demonstrating that electrical stimulation not only enhances fibroblast proliferation and collagen deposition but also activates the PI3K/AKT pathway to promote vascular regeneration. This innovative strategy, which converts biomechanical stimuli into therapeutic electrical signals, provides a crucial theoretical foundation and technological pathway for developing novel self-powered wound treatment systems.

### 3.7 FDA-approved electrical stimulation devices

In the 2017 study by Bikson et al. (2018), FDA-approved TENS devices (primarily Class II medical devices cleared through the 510(k) premarket notification process) were categorized into two main applications: GZJ devices for pain management (e.g., arthritis, migraine treatment) and NFO devices for cosmetic purposes (e.g., facial rejuvenation). The study noted that these devices could deliver a maximum output current of up to 37.6 mA in head and facial applications (exemplified by the Rejuvenique device) and demonstrated good clinical tolerance, with common side effects limited to transient skin irritation (e.g., erythema resolving within 20 min, as reported in studies on the BMR Face device). A representative study, such as Kavanagh et al.’s 2012 clinical trial on facial rejuvenation, showed that using a TENS device with a peak current of 35 mA (5 sessions per week, 20 min per session, over 12 weeks) resulted in no significant adverse events in the treatment group (n = 56) compared to the control group (n = 52). Notably, although the electrical parameters of TENS devices (e.g., current density of 46.4 mA/cm^2^ for the Rejuvenique OTC device and 2.37 mA/cm^2^ for the prescription device Cefaly) were significantly higher than those of traditional tES technologies, their long-term clinical use had validated their safety.

The 2023 study by Bikson et al. (2023) further clarified that the FDA has established stringent electrical output safety standards for TENS devices (Class II medical devices). Key restrictions include: a charge quantity formula based on phase duration *t* (milliseconds), *Q* = 20 + 28*t* mC (measured at 50% phase amplitude), an average current ≤ 10 mA, depolarization phase duration ≤500 ms, and a DC current ≤ 100 μA under fault conditions. Additionally, the FDA mandates that the RMS electrode current density must be ≤ 2 mA/cm^2^ and the average power density ≤0.25 W/cm^2^. Furthermore, the FDA specifies multiple safety measures, covering dynamic output control, open/short-circuit protection for electrodes, power stability management, and biocompatibility requirements for electrode materials. These measures, enforced through the 510(k) review process, ensure end-to-end safety from device design to clinical application.

### 3.8 Optimization of electrical stimulation parameters: the influence of intensity, frequency, and waveform on wound healing

The study by Li et al. (2020) systematically analyzed the effects of different electrical stimulations on wound healing: Direct current (DC) leads to voltage concentration due to high epidermal resistance but shows limited efficacy and may even cause necrosis, as observed in diabetic rats where it accelerated wound contraction without significantly improving inflammation. Monophasic pulses (SP), owing to better voltage distribution and the ability to promote α-SMA and TGF-β1 secretion, significantly enhanced healing efficacy. In contrast, biphasic pulses (BP) exhibited poorer therapeutic effects due to charge cancellation, though adjusting frequency (≤187 Hz) and duty cycle (e.g., <50.6%) could improve outcomes. The key factor, charge quantity, has an effective range of 250–500 μC/s in humans and 75–90 μC/s in rats, with the DC component (UDC) optimally ranging between 0.49 and 0.7 V, though experiments showed its safe range could extend to 0.4–1.22 V. These findings provide critical insights for optimizing electrical stimulation therapy.

Lin et al. (2025) developed a hydrogel-based electronic wound dressing patch that enables precise drug release in diabetic wound healing by optimizing electrical stimulation parameters. The study found that applying a −5 V DC voltage allowed the PEDOT:CHC/silk hydrogel to release 60% of the drug within 1 h, far surpassing other samples. However, increasing voltage intensity exacerbated continuous drug outflow. Periodic monophasic constant potential stimulation (−3 V for 2 min) led to burst release with diminishing efficiency, whereas biphasic alternating current (AC) stimulation produced a controllable stepwise release, accelerating responsive release while suppressing continuous diffusion. Experiments demonstrated that continuous biphasic AC cycles were the most effective strategy for regulating ibuprofen release, enabling on-demand drug delivery by adjusting voltage parameters. Thus, selecting the appropriate electrical stimulation mode (particularly biphasic AC) is crucial for optimizing drug release and promoting wound healing.

Xu et al. (2022) found that non-contact electrical stimulation (NCES) at specific intensities significantly enhances wound healing. In HaCaT cells, NCES at 53 and 76 mV mm^-1^ boosted cell viability on day 1 and consistently promoted migration and proliferation on days 3 and 5. For human dermal fibroblasts (HDFs), 53 mV mm^-1^ was most conducive to migration and uniform healing, while 76 and 104 mV mm^-1^ promoted vertical cell alignment. In THP-1 and macrophages, NCES at 53 mV mm^-1^ upregulated M2 macrophage markers, optimizing immunomodulation. Animal experiments showed that NCES at 54 mV mm^-1^ consistently accelerated healing, reduced scarring, and improved collagen alignment. In summary, NCES at 53–54 mV mm^-1^ significantly enhances wound repair by regulating cell behavior, immune response, and tissue remodeling.

## 4 Materials

Wound healing is a complex biological process that involves multiple stages. Electrical stimulation has gained attention as an adjunct therapy to promote wound closure. The materials used in electrical stimulation devices play a vital role in determining the efficacy and safety of the treatment. There are more and more types of wound dressing materials on the market, but chronic wounds do not respond quickly to traditional dressings (Cheah et al., 2021). There is an urgent need to find new dressings that can play a positive role in electrically stimulating wound healing. After continuous exploration by scientists, as shown in Table 2, the current materials used in academia for electrically stimulated wound healing mainly include conductive polymers, carbon-based materials, hydrogels, etc.

**TABLE 2:** The comprehensive review of materials utilized in ES for enhanced wound healing.

Material	Preparation	Intensity/Duration/Frequency of ES	Animal model	Effect of wound healing	Ref.
Double-conductive hydrogel electrode	Main chain composed of poly(N-isopropylacrylamide-co-acrylamide) (NIPAM-AAm) copolymer, forming a double-network structure with conductive polymer PEDOT:PSS	Frequency: 13.56 MHz; Voltage: 0–2 V	6 mm diameter wound in C57BL/6 mice	Wound closure rate increased by 25%; collagen deposition increased by 50%; microvascular density improved by 60%; bacterial colony count reduced by 50%	Jiang et al. (2023)
TENG	Top layer: flexible triboelectric layer (PCL-r-PU); middle layer: PEDOT:PSS electrode; bottom layer: PAV bioadhesive with Mo electrode	AC electric field: 20 kHz	*In vitro* model: 5 mm diameter porcine intestinal defect; *in vivo* model: 5 mm × 3 mm liver incision and 1.5 cm × 0.5 cm skin incision in rats	Hemostasis within 5 s (blood loss reduced by 82%); skin wound healed by day 3; fibroblast proliferation rate increased by 84%	Meng et al. (2023)
Fully implantable wireless tactile sensing artificial skin (WTSA)	Composite hydrogel crosslinked at low temperature (collagen/fibroin + genipin); electrochemically deposited Au-black nanoparticles; PDMS/Parylene C/Al_2_O_3_ multilayer encapsulation	Frequency range: 0.53–58.8 Hz (pressure: 30.2–96.4 kPa); output signal: sawtooth pulses (frequency correlated with pressure)	10 mm diameter full-thickness skin defect in 6-week-old SD rats	Closure rate improved by ≥ 20%; collagen density increased by ∼50%; loricrin expression elevated 3.2-fold	Kang et al. (2024)
3D-printed ZnO nanoparticle-modified PVDF/sodium alginate (SA) piezoelectric hydrogel scaffold (ZPFSA)	Composite hydrogel with 8% PVDF, 6% SA, and 0.5% ZnO; dissolved in DMF/water mixture and Ca^2+^ crosslinked	*In vitro* output current: ZPFSA-0.5 group ±1.10 μA (expansion + friction: ±1.29 μA); *in vivo* output current: initial ±0.386 μA (day 0), decreasing to ±0.12 μA (day 14)	Full-thickness skin defect (1 × 1 cm) in SD rats	Healing rate increased by 28.2%; collagen content increased by 37.8%; antibacterial efficacy against MRSA improved by 528%	Liang et al. (2022)
Multilayer stacked electret	Fluorinated ethylene propylene (FEP) films charged via corona charging system	Surface potential: 3400 V (7-layer stack)	9 mm diameter full-thickness skin defect in 8-week-old female C57BL/6 mice	COL1A1 protein expression reduced by 90%; α-SMA-positive signals decreased by 44%; scar area reduced by 60%; collagen density decreased by 33%	Kim et al. (2024a)
Photovoltaic microcurrent hydrogel dressing	Methacrylated hyaluronic acid (HA-MA-DA) modified with dopamine and in situ-synthesized Ag nanoparticles (HD-Ag_2_)	Electric field strength: 100 mV mm^-1^	10 mm diameter infected full-thickness skin defect in high-fat diet/STZ-induced type 2 diabetic mice	Wound closure rate increased by 21.3% vs. control and 14.1% vs. HD-Ag_2_ group; collagen deposition reached 320% of control; macrophage ratio elevated 2.5-fold	Hu et al. (2024)
lectroconductive polymer-based electro-mechanical synergistic dressing	PPy-coated carbon cloth (CC) electrode layer with bacterial cellulose (BC) hydrogel electrolyte layer	Voltage: 1 V; duration: 10 min; current density: 2 mA/cm^2^	10 mm diameter infected full-thickness skin defect in 6-week-old male BALB/c mice	Bacterial inhibition rate improved by 13.1%; wound area reduced by 39.2% at day 3; complete healing by day 14; collagen deposition increased by 45%	Wang et al. (2024b)
Flexible hydrogel	PANI as conductive phase, SHA as macromolecular dopant, embedded in polyacrylamide (PAM) matrix via *in situ* polymerization	3 V DC, 1 h/day	Chronic infected wound model in diabetic SD rats	Wound closure rate increased by 21%; epithelial thickness doubled; collagen deposition rate increased by 100%; granulation tissue width reduced by 65%	Wu et al. (2021)
Light-controlled conductive polymer dressing	Polyaniline (PANI) doped with visible light-activated photoacid (MEH), electrochemically deposited on carbon cloth	Light intensity: 14.29 mW/cm^2^	8 mm diameter full-thickness dorsal skin defect in male SD rats	Wound area healed by 81% at day 7; complete healing at day 14; collagen deposition reached 61.8% of normal skin	Yin et al. (2023)
Wearable triboelectric stimulator (WTS) with flexible triboelectric nanogenerator (F-TENG) and tribo-responsive drug-delivery hydrogel (TR-DDH)	Laser-engraved conical silicone mold; PPy/curcumin nanoparticles (PPy/CUR NPs); PVA-phytic acid (PVA-PA) hydrogel	F-TENG parameters: open-circuit voltage 240 V, short-circuit current 4 μA, charge transfer 40 nC; maximum power output: 100 μW at 40 MΩ	6 mm diameter infected dorsal wound in nude mice	Full-thickness wound healing rate: 28.5%; bacterial infection healing rate improved by 85.2%; *S. aureus* inhibition rate increased by 41.6%	Qin et al. (2024)
Ionic triboelectric nanogenerator (iTENG) patch (electro-mechanical synergistic dressing)	Bilayer PDMS film sandwiching gelatin nanocapsule (GeI NCs) hydrogel; 4 × 4 perforated array on PDMS surface	Output voltage: diabetic gait (early: 54.11 mV; mid: 60.87 mV; late: 30 mV)	8 mm diameter full-thickness skin defect in diabetic mice	Collagen deposition rate increased by 78% vs. control; vascular density elevated by 499%	Zhang et al. (2024d)
Sulfonated hyaluronic acid/graphene-based hydrogel (siHA/G-GM)	Sulfonated hyaluronic acid (siHA)-modified graphene uniformly dispersed in gelatin methacrylate (GelMA)	Endogenous electric field (peak wound current: siHA/G-GM group 1,367.78 nA vs. GM group 3.25 nA)	0.9 cm diameter full-thickness dorsal skin defect in rats; 10 mm defect in rabbit ear	Wound closure rate improved by 12.8%; collagen I/III ratio reduced to 1.2; scar index decreased by 42.3%; collagen I/II ratio increased to 78%	Zhong et al. (2024)
PPTZ hydrogel (PVA/PA/TA/ZnCl_2_ composite hydrogel)	Dual-network: first network (PVA/PA/TA via H-bonding and Zn^2+^ coordination); second network (PVA crystalline domains)	Pulsed current: 1 Hz, 8 mA, 30 min/day (15 min polarity reversal)	8 mm diameter infected full-thickness skin defect in STZ-induced diabetic mice	Antibacterial rate >99%; healing rate increased by 72.8%; α-SMA expression elevated by 201%	Zheng et al. (2024)
Conductive hydrogel (PVNP-SP)	Porous structure with spirulina-loaded pores	*In vitro*: 1 Hz, 0.05 mA, 30 min (15 min polarity reversal); *in vivo*: 1 Hz, 8 mA, 30 min (15 min polarity reversal)	8 mm diameter full-thickness skin defect in STZ-induced diabetic mice	Wound area reduced to 3.9% at day 10; collagen density increased 2.1-fold; microvascular density elevated by 452%	Liu et al. (2024)

### 4.1 Conductive polymers (CP)

A CP is a polymer with a conjugated π-electron system and the ability to conduct electricity. In the field of electrical-stimulation-assisted wound healing, CPs can be used as electrode materials or carriers for active ingredients, etc. It can effectively conduct current when an electrical stimulation is applied and may also have characteristics such as biocompatibility, which helps to promote cell activities, proliferation, etc., at the wound site, thereby accelerating wound healing. They have adjustable conductivity and good biocompatibility. They can be designed to have specific electrical and mechanical characteristics according to needs, making them suitable for wound healing applications.

However, a single application CP is impractical, its mechanical properties are poor, its rigid structure makes it highly intolerant, and it is highly sensitive to moisture and air (Acosta et al., 2022). Long-term implantation also carries the risk of chronic inflammation. Therefore, scientists have found that when CP is mixed with other materials such as silk (Gh et al., 2017), collagen (Qin et al., 2020), gelatin, or prepared into hydrogels based on CPs (Stejskal, 2017), the defects of CP can be effectively improved, and hybrid CP has become a research hotspot at present.

CP materials are widely used in nerve regeneration and bone repair and are capable of controlling the growth of cells and tissues by promoting cell proliferation, simulating electronic or ionic conductivity, and medicating the flow of currents (Dong et al., 2017; Gilmore et al., 2009; Aznar-Cervantes et al., 2017; Qi et al., 2018). At present, the commonly used CPs on the market include polypyrrole (PPy), polythiophene (PT) and its derivatives, polyaniline (PANI), such as poly(3,4-ethylenedioxythiophene) (PEDOT).

#### 4.1.1 Polypyrrole (PPy)

PPy, with its simple synthesis process and excellent electrical conductivity, is one of the most widely used CPs (Li et al., 2019). However, the application of PPy is restricted by its poor mechanical and processing properties. Fortunately, the low mechanical problem of PPy can be overcome by combining the PPy with other flexible polymers to construct composite conduction material for various biomedical applications (Tang et al., 2022; Huang et al., 2022).

**FIGURE 6:**
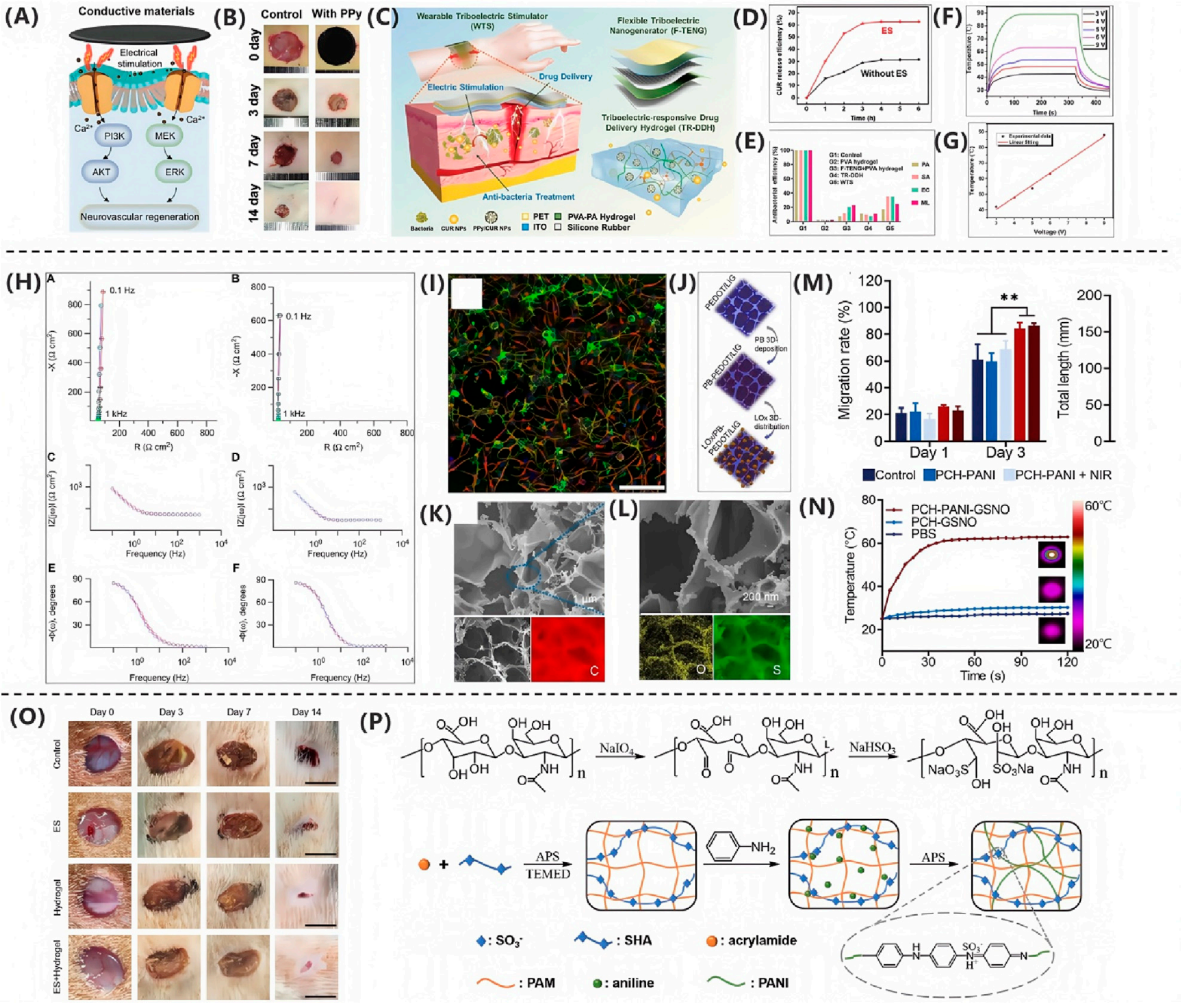
**(A)** Three electrical stimulation strategies for tissue repair. **(B)** Conductive hydrogels enhance regeneration via Ca^2+^-PI3K/AKT/ERK (Kim HS. et al., 2024). Copyright 2022, Adv Healthc Mater. **(C)** Triboelectric stimulator drives PPy/CUR NP release. **(D)** ES group achieved >60% CUR release. **(E)** WTS group showed >95% antibacterial rate (Qin et al., 2024). Copyright 2024, Adv Healthc Mater. **(F)** SA/rGO/PPy textile heated rapidly (3–9 V). **(G)** Temperature followed Joule’s law (Bi et al., 2021). Copyright 2021, Chem Eng J. **(H)** PEDOT:PSS maintained low impedance. **(I)** PEDOT:PSS substrates promoted neural precursors (Collazos-Castro et al., 2010). Copyright 2021, Biomaterials. **(J)** Schematic diagram of PB deposition and oxygen loading on porous PEDOT/LIG. **(K)** SEM image of PEDOT/LIG. **(L)** EDS spectrum of PEDOT/LIG (Meng LY. et al., 2021) . Copyright 2021, ACS Appl Mater Interfaces. **(M)** Migration rate of L929 cells treated with PCH-based nanofiber membrane on days 0, 1, and 3. **(N)** Temperature changes of PCH-PANI-GSNO, PCH-GSNO, and PbS under NIR irradiation (Xie et al., 2024). Copyright 2024, J Nanobiotechnol. **(O)** Representative wound healing images. **(P)** Preparation process of PSP hydrogel (Wu et al., 2021). Copyright 2021, ACS Appl Mater Interfaces.

Kim HS. et al. (2024) demonstrated that extracellular matrix-mimicking conductive hydrogels significantly accelerate diabetic wound healing through electroactivity. The core mechanism involves the conductive polymer network mimicking the skin’s electrical conductivity. As illustrated in Figure 6A, this material enhances endogenous electric field signaling, markedly elevating intracellular Ca^2+^ concentrations in endothelial and neural cells. This subsequently activates phosphorylation cascades in PI3K/AKT and MEK/ERK signaling pathways, driving angiogenesis and neural regeneration. Animal studies validated therapeutic efficacy as shown in Figure 6B, with diabetic rat models revealing significantly superior wound contraction rates in the conductive hydrogel-treated group versus controls, achieving near-complete re-epithelialization by day 14.

Qin et al. (2024) developed a WTS integrating a flexible triboelectric nanogenerator (F-TENG) and a triboelectric-responsive drug-delivery hydrogel (TR-DDH) for efficient treatment of bacterially infected wounds. Figure 6C illustrates the overall design architecture of WTS, wherein the F-TENG harnesses biomechanical energy for electrical stimulation while the TR-DDH serves as a wound-contact electrode ensuring stable signal transmission. The TR-DDH, incorporating polypyrrole/curcumin nanoparticles (PPy/CUR NPs), utilizes polypyrrole valence reduction mechanisms to trigger controlled drug release under electrical stimulation. Figure 6D confirms a curcumin release efficiency reaching 60% within 3 h, significantly optimizing bioavailability. *In vivo* infection models demonstrated that WTS synergistically facilitated the M1-to-M2 macrophage phenotypic transition, suppressed expression of pro-inflammatory cytokines IL-1β and TNF-α, and accelerated collagen regeneration and re-epithelialization. Figure 6E quantifies its antibacterial efficiency exceeding 60%, markedly superior to monotherapy, ultimately achieving high-quality tissue repair.

As demonstrated by Bi et al. (2021), the SA/rGO/PPy composite textile synthesized via thermal compression reduction and *in situ* polymerization primarily functions as a flexible heating module. As shown in Figures 6F,G, this material achieves precise temperature control between 40 °C and 90 °C under low voltages of 3–9 V. Its inherent flexibility ensures biocompatibility with biological tissues, thereby providing a smart bandage platform for controllable thermal stimulation and drug delivery that facilitates closed-loop regulation of the wound microenvironment.

#### 4.1.2 Polythiophene (PT) and its derivatives

PT is a conductive polymer. It is polymerized from thiophene monomers and has a conjugated π-electron system. This conjugated structure endows polythiophene with unique electrical properties, such as the ability to adjust its conductivity by means of doping, etc. However, due to its conductivity, instability in air, susceptibility to reduction to its intrinsic state, and difficulty in processing, polythiophene is not as widely used as its derivative PEDOT (poly (3,4-ethylenedioxythiophene)).

According to the research by Collazos-Castro et al. (2010), electrochemical interface modification technology enables customized regulation of PEDOT:PSS materials to meet the sequential requirements of wound healing. The study employed electrical stimulation with a current density of 0.1 mA cm^-2^ and negative pulses of 100 ms, achieving dynamic modulation of surface bioactivity through the reversible desorption of a heparin layer. As shown in Figure 6H, the electrode interface impedance remained stably below 200 Ω cm^-2^ under cell culture conditions, ensuring the safety of electrical stimulation. Figure 6I further demonstrates that the heparin-basic fibroblast growth factor composite layer specifically inhibits neuronal development while significantly promoting the proliferation and migration of NG2-positive precursor cells. This effect was validated by vimentin immunofluorescence and can be applied to the proliferative phase of wound healing to facilitate granulation tissue formation. By leveraging the synergistic effects of molecular self-assembly and electrochemical signals, this study provides an innovative theoretical framework for the development of intelligent electroactive dressings.

PEDOT (poly (3, 4-ethylenedioxythiophene)) is a derivative of polythiophene. It has high conductivity and good stability and is currently widely used in wound-healing dressings. Poly (3, 4-ethylenedioxythiophene) (PEDOT) presents a stable andconductive “doped” state, which is beneficial for serving as an electricallyconductive matrix for *in situ* embedding of a catalyst (Fan et al., 2022). Such as Meng LY. et al. (2021) developed a polythiophene derivative-enhanced PEDOT/laser-induced graphene heterostructure, fabricating a three-dimensional conductive network via *in situ* electrochemical polymerization as depicted in Figure 6J. This architecture substantially enhances the electrode’s mechanical stability and electrochemical interfacial charge transfer efficiency. Its hierarchically porous framework, detailed in Figures 6K,L, serves as an efficient biological carrier, achieving high-loading immobilization of lactate oxidase and Prussian blue within the three-dimensional space to form a compact heterointerface. The flexible skin patch constructed from this heterostructure conformally adheres to dynamic wound tissues, enabling broad linear detection of lactate in the wound microenvironment across a 0–18 mM range with a sensitivity of 2.23 μA mM^-1^. This dual-channel patch system simultaneously monitors glucose and lactate levels, establishing a novel strategy for continuous tracking of critical metabolic biomarkers in smart wound dressings.

For example, Jian-Jr Lee’s team fabricated gelatin-methacrylate (GelMa) hydrogels with different concentrations using poly (3,4-ethylenedioxythiophene) (PEDOT): polystyrene sulfonate (PSS), which enhanced the scaffold strength and conductivity and was able to enhance the efficiency of electrical stimulation in promoting wound healing (Lee et al., 2022). Wang et al. (2020) developed a novel electroactive hydrogel of regenerated bacterial cellulose/polypyrrole/carbon nanotubes (rBC/PPy/CNT) that promotes cell proliferation through the methods of cellulose dissolution and physicochemical cross-linking to facilitate wound healing using electric fields (EF), The hydrogel was characterized by field emission scanning electron microscopy (FESEM), Fourier transform infrared spectroscopy (FTIR), X-ray diffraction (XRD), thermogravimetric analysis (TGA), conductivity, mechanical and swelling tests. The results showed that PPy and CNTs were successfully deposited in the rBC/PPy/CNT hydrogel, which exhibited excellent thermal stability, mechanical strength, recoverability, swelling ability, and its electrical conductivity was 107 times higher than that of rBC. It is a material with outstanding properties.

#### 4.1.3 Polyaniline (PANI)

Polyaniline has become one of the most commonly used CPs in the field of electrical stimulation of wound healing.

At present, the antibacterial properties of materials used for electrical stimulation wound healing have become the main reason restricting its development. Currently, the commonly used cationic antibacterial agents have some limitations in biomedical applications, such as toxicity (such as Ag^+^) and high cost (such as specially designed antimicrobial peptides) (Fang et al., 2018; Wei et al., 2021). Therefore, in the study of Xie et al. (2024) demonstrated that polyaniline-based composite nanofiber membranes significantly accelerate healing of infected diabetic wounds through near-infrared-responsive photoelectrical stimulation. As shown in Figures 6M,N, the polyaniline component confers exceptional photothermal conversion properties, rapidly elevating temperature to 58 °C within 30 s under 808 nm near-infrared irradiation. This thermal effect directly eliminates pathogens while simultaneously triggering burst release of nitric oxide from S-nitrosoglutathione, synergistically enhancing antibacterial efficacy. Crucially, the conductive microenvironment established by polyaniline markedly accelerates fibroblast migration by modulating cellular electrical signaling. An 86.5% cell migration rate on day 3 in the combined treatment group, representing a 41% increase over controls. This effect originates from nitric oxide-mediated upregulation of vascular endothelial growth factor signaling pathways, driving angiogenesis and collagen deposition. In diabetic rat models with infected wounds, this material achieved complete re-epithelialization within 14 days through a photoelectric-chemical cascade conversion mechanism. Notably, epidermal thickness increased by 56% and neovascular density surged by 216%, confirming that polyaniline-based materials provide an efficient therapeutic strategy for drug-resistant infected wounds by mimicking endogenous electrical stimulation microenvironments.

Wu et al. (2021) this study developed a light-driven wound dressing based on long-lived photosensitive acid-doped polyaniline composites. Figure 6O demonstrates an 81% wound closure rate after 14 days of visible-light irradiation in the PANI/MEH dressing group, significantly surpassing the control group. This superior efficacy originates from multiple synergistic mechanisms: the photocurrent effectively mimics endogenous electric fields, guiding cell migration and proliferation via electrotaxis to promote fibroblast activation and collagen deposition; MEH-established localized weakly acidic environments suppress microbial infection; concurrently, the material’s superhydrophilicity maintains a moist wound microenvironment, collectively accelerating re-epithelialization and tissue remodeling. The exceptional healing outcomes correlate closely with its outstanding photoelectrical properties. As shown in Figure 6P, the PANI/MEH composite achieves a photocurrent density of 60 μA cm^-2^ at pH 4.0, representing a 2.5-fold enhancement over conventional PANI materials. This performance improvement stems from MEH-mediated sustained proton release, which triggers reversible redox reactions along the polyaniline backbone to generate stable photocurrent.

The study by He et al. (2020) also confirmed the antibacterial properties of PANI as a material. They combined the excellent mechanical properties of PCL with the multifunctionality of quaternized chitosan grafted polyaniline (QCSP), and prepared a series of antibacterial, antioxidant, and electroactive nanofiber membranes by electrospinning PCL, QCSP polymer solution, and nanofiber membrane (NFM). It exhibits good water absorption ability, which can fully absorb the exudate of the wound. At the same time, during the inflammatory stage of wound healing, neutrophils, white blood cells, and monocytes are attracted to the wound site by bioactive media, and then attack microorganisms and external debris through phagocytosis. This process will lead to excessive production of free radicals, including superoxide anions, hydrogen peroxide, and hydroxyl anions (Agyare et al., 2013). PCL/QCSP NFM wound dressings have demonstrated excellent scavenging ability against these excess free radicals, demonstrating their broad application prospects in the field of wound healing.

### 4.2 Graphene and its derivatives

Graphene wound dressings are gradually attracting attention in some research and clinical applications. Graphene oxide (GO) is an oxidized form of graphene with single-atomic layers that exhibit excellent conductivity and high chemical stability (Yu et al., 2023). GO exists as a two-dimensional lattice comprising a unique monolayer of sp^2-^and sp^3-^hybridized carbon atoms. Previous studies confirmed the ability of GO to promote cell proliferation and adhesion (Fu et al., 2017). and that conductive polymer materials containing GO can strengthen the ES, thereby accelerating wound healing (Khan et al., 2015; Senthil et al., 2018). Additionally, GO mediates the formation of extracellular superoxide compounds that can inhibit bacterial activity and reduce the risk of infection (Zhao et al., 2018). Moreover, dispersed GO nanoparticles exhibit low viscosity and cytotoxicity at high concentrations, thereby making them good candidates for local administration. The dispersed nanoparticles can also be converted into membrane materials to prolong their residence time on the skin surface and prevent biotoxicity (Rauti et al., 2016).

**FIGURE 7:**
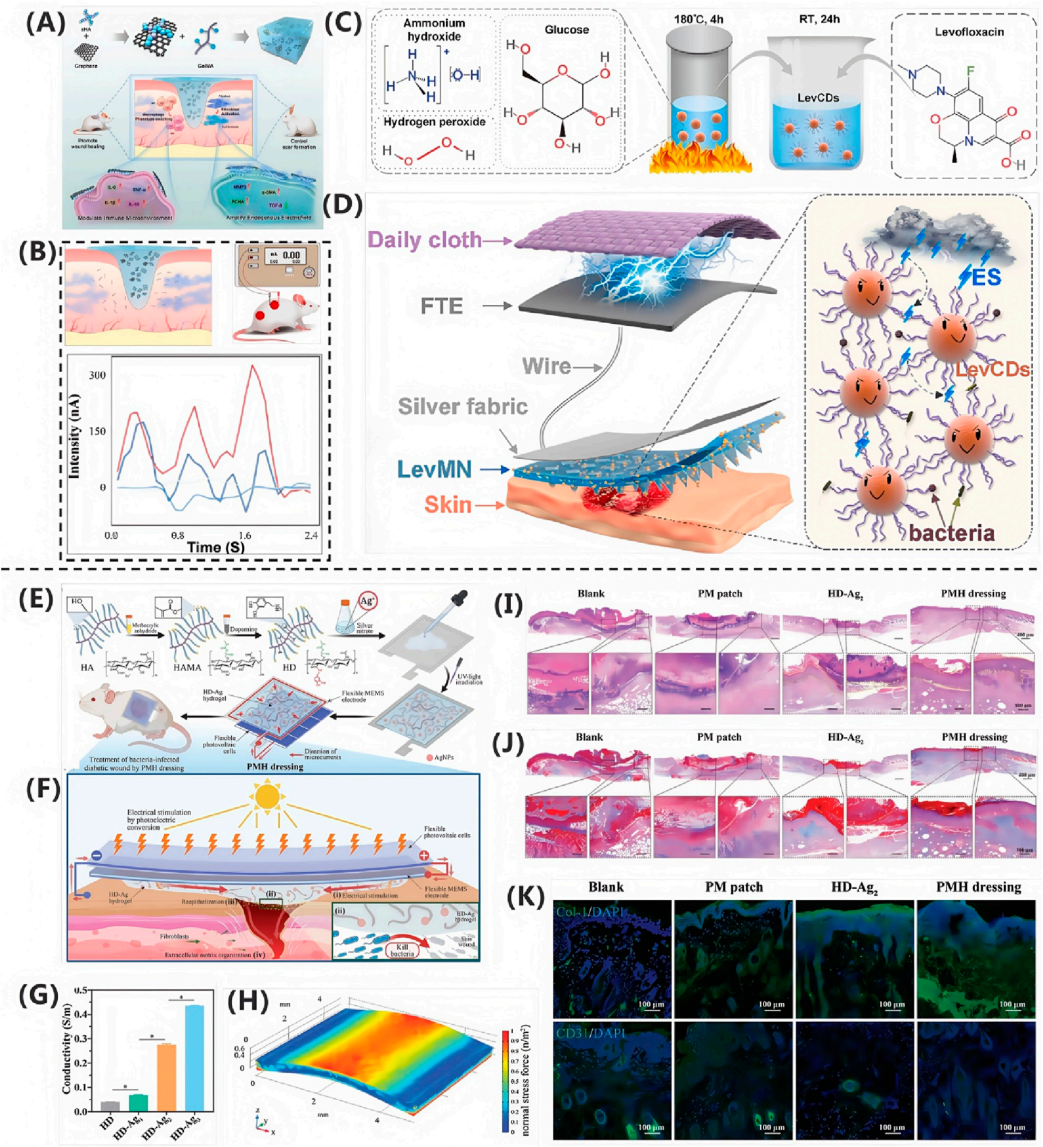
**(A)** siHA/G complex enhances electric fields for scarless healing. **(B)** Increased M2 macrophages and reduced TNF-α (Zhong et al. (2024)). Copyright 2024, Chem Eng J. **(C)** Polypyrrole substrate promotes neural regeneration. **(D)** Triboelectric microneedles enable ES + drug delivery (Zhang SG. et al., 2024). Copyright 2024, Chem Eng J. **(E)** AgNP-hydrogels for diabetic wounds. **(F)** PMH hydrogel’s multimodal action. **(G)** HD-Ag_3_ achieved 0.35 S·m^-1^ conductivity. **(H)** Structural integrity under compression. **(I–K)** PMH improved healing outcomes (Hu et al., 2024). Copyright 2024, Adv Sci.

Zhong et al. (2024) demonstrated scarless healing of infected wounds using sHA/G-GM conductive hydrogels. The underlying mechanism, systematically illustrated in Figure 7A, involves the sulfated hyaluronic acid component within the material specifically binding IL-4 via sulfonate groups. This binding activates the STAT6 pathway in macrophages, promoting their polarization from the M1 to the M2 phenotype. Concurrently, the graphene network enhances tissue conductivity, amplifying endogenous electrical signals to coordinately regulate fibroblast migration and collagen deposition. Flow cytometry data in Figure 7B confirmed a significantly elevated proportion of CD206+ M2 macrophages within the treated wounds compared to the control group, accompanied by suppressed expression of the pro-inflammatory cytokine TNF-α. These findings validate the sHA-mediated immune remodeling function. The synergistic actions of these mechanisms promote angiogenesis and epithelial regeneration, ultimately culminating in organized collagen arrangement and functional wound healing.

Zhou et al. (2024) developed a conductive polyacrylamide/sodium alginate cross-linked hydrogel composite containing reduced graphene oxide (PAA-SA@rGO). This composite material has antibacterial, anti-inflammatory and antioxidant properties and can serve as a bridge to turn the “short circuit” at the injured site into a “complete circuit”, thereby prompting fibroblasts near the wound site to secrete growth factors and accelerate tissue regeneration. Meanwhile, the PAA-SA@rGO hydrogel can effectively seal the wound to form a barrier, demonstrating antibacterial and anti-inflammatory properties and preventing the invasion of foreign bacteria.

### 4.3 Carbon-based materials

Carbon-based materials such as carbon nanotubes and graphene have high conductivity and mechanical strength. They can be used to make flexible electrodes or incorporated into wound dressings for electrical stimulation. Carbon has high electrical conductivity, good mechanical properties and a large specific surface area. A large specific surface area helps to adsorb some bioactive molecules, etc., and high electrical conductivity can ensure effective electrical-stimulation transmission, thus playing a role in the wound-healing process, such as regulating the electrical environment of cells at the wound site and promoting cell migration and proliferation.

#### 4.3.1 Carbon nanotubes (CNTs)

CNTs have various applications in electrically-stimulated wound healing. Their excellent electrical conductivity can conduct current, change the electric field environment of the wound, promote the proliferation and migration of fibroblasts and keratinocytes, and contribute to wound repair. Their good mechanical properties can enhance the mechanical strength of wound dressings or scaffolds, providing a stable physical environment for cell growth and tissue reconstruction (Feng et al., 2015). They can load bioactive substances such as drugs and growth factors, and achieve controlled release under electrical stimulation, regulating cell differentiation and tissue regeneration, as well as antibacterial and anti-inflammatory effects. In addition, the antibacterial properties of some carbon nanotubes can reduce the risk of wound infection, which is beneficial to wound healing.

Zhang SG. et al. (2024) developed a wearable self-powered microneedle system based on conductive levofloxacin-carbon quantum dot composites for treating infected wounds. Figure 7C confirms the uniform nanostructure and lattice characteristics of LevCDs, endowing them with excellent electrical conductivity. Figure 7D demonstrates the synthesis of this agent via covalent conjugation strategy, achieving dual-functional integration of antibacterial activity and conductivity. The microneedle patch delivers LevCDs into deep wound tissues while simultaneously transmitting electrical stimulation generated by a triboelectric nanogenerator. *In vitro* experiments revealed that electrical stimulation significantly promotes fibroblast migration and proliferation, with LevCDs exhibiting minimum inhibitory concentrations of 1.875 μg/mL and 0.469 μg/mL against Gram-positive and Gram-negative bacteria, respectively. In infected mouse models, this system accelerated collagen deposition and epithelial regeneration via electrical stimulation, synergizing with the reactive oxygen species-mediated bactericidal action of LevCDs. This combined approach achieved a 95.6% wound healing rate within 12 days, significantly outperforming monotherapy groups. The technology establishes a novel synergistic electrostimulation-drug delivery strategy for infected wound management.

### 4.4 Hydrogels

Hydrogel dressing is a new type of medical dressing, which is cross-linked by hydrophilic polymer. Hydrogel dressings have a structure similar to extracellular matrix, and have good biocompatibility. They can absorb wound exudates and provide a humid environment around the wound, so they are widely used in the treatment of skin wounds (Zhou et al., 2019; Zhang et al., 2019). At the same time, hydrogel dressings can also be used as carriers of bioactive factors or antibacterial drugs to improve the skin healing rate (Tahneh et al., 2022). However, due to the poor conductivity and lack of antibacterial properties of individual hydrogels, it is impractical to use hydrogels alone in wound healing. At present, the widespread practice is to introduce conductive polymers, carbon based materials, etc., into hydrogels, which has become a research hotspot in the field of electrostimulation wound healing.

The PMH dressing developed in this study features a sandwich structure with a core comprising a flexible organic photovoltaic module, a microelectromechanical systems electrode, and an HD-Ag_2_ conductive hydrogel interface. As illustrated in Figures 7E–K, the hydrogel incorporates *in situ* synthesized silver nanoparticles via photoreduction, endowing it with both biocompatibility and antibacterial activity. In an infected diabetic wound model, this dressing generates biomimetic electrical stimulation through photoresponsive mechanisms, mimicking the direction of physiological injury currents. Experimental results confirm its triple-action healing acceleration mechanism: first, the HD-Ag_2_ hydrogel effectively eliminates MRSA and PAO1 pathogens, exhibiting an inhibition zone diameter of 8.2 ± 0.3 mm; second, it downregulates pro-inflammatory cytokines TNF-α and IL-1β while promoting M2 macrophage polarization, thereby shortening the inflammatory phase; finally, it activates fibroblast migration and upregulates collagen deposition by 320%, concurrently enhancing extracellular matrix reorganization. *In vivo* studies demonstrate a 97% wound closure rate within 22 days, significantly surpassing the control group (Hu et al., 2024).

Here, Xu RC. et al. (2024) Proposed a porous hydrogel with gas-solid contact separation triboelectric (gshl) as wound dressing with reference to. Their synthesis process began with the preparation of a solution containing deionized water, into which lithium chloride and sodium dodecyl sulfate were dissolved. After the initial reagent was completely dissolved, dodecyl methacrylate was introduced into the solution, which improved the structural integrity and mechanical properties of the hydrogel. Then, acrylamide was added as monomer of self-polymerization reaction and potassium persulfate as initiator of polymerization reaction. The addition of N, N, n’, n’- tetramethylethylenediamine to catalyze the polymerization ensures the rapid and effective crosslinking of hydrogels. During the whole reaction, the mixture was stirred vigorously to promote the formation of uniform porous structure. After the preparation, in order to evaluate the medical application potential of gshl as wound dressing, they evaluated several important indicators, including expansion rate, tensile strength, tensile strain and stability. They showed good swelling rate, which also provided good conditions for the wound not to be exposed. At the same time, it also had good tensile strength and stability. As a wound dressing, it was undoubtedly excellent.

## 5 Electrical stimulation effect

ES, as an adjuvant therapy, can significantly enhance treatment efficacy when combined with other therapies. Xuan Qin’s research team has developed an innovative wearable triboelectric stimulation device (WTS) that effectively enhances the healing of infected wounds. This system combines flexible triboelectric nanogenerator (F-TENG) technology with intelligent hydrogel (TR-DDH), enabling both electrical stimulation and on-demand drug delivery. Animal studies demonstrated that WTS achieves superior healing outcomes compared to single treatment approaches, significantly accelerating wound recovery while reducing inflammation (Qin et al., 2024).As a promising adjuvant therapy, electrical stimulation has antibacterial effects, promotes cell migration and proliferation, accelerates angiogenesis, promotes proper deposition of collagen, and anti-inflammatory effects, which play a crucial role in wound healing (Fernández-Guarino et al., 2023).

### 5.1 Antibacterial effect

Electrical stimulation techniques have been shown to be effective in reducing bacterial load in wounds while protecting host cells from damage. In the study of Marziyeh Jannesari (Jannesari et al., 2023) a TENG composed of PPy-GO composite material was successfully developed, which showed excellent electrical output performance and significant bactericidal activity. In order to further explore its antibacterial mechanism, *Staphylococcus aureus* was used as the representative model of gram-positive bacteria, and the antibacterial properties of the prepared TENG electrode were studied in detail. In order to quantitatively evaluate its antibacterial performance, optical-density (DO600) measurements and colony-forming unit-counting methods were used to monitor the inhibition of bacterial growth. The results of experimental significant destructive changes were observed in the incubation process of bacteria on the electrode under the condition of no electrical stimulation. However, when the tapping time was extended from 10 min to 25 min, the bactericidal performance of the electrode was significantly improved, and the bactericidal rate was increased from about 25% to about 75%. This phenomenon can be attributed to the triboelectric effect produced under tapping conditions. According to the morphological observation of bacteria, on the carbon electrode treated without electric field stimulation by TENG, the originally smooth and complete *S. aureus* became fuzzy and rough after modification by PPy and PPy-GO layers. In the absence of electric field stimulation of TENG, no rupture or collapse of bacterial envelope or cell membrane was observed. However, the bactericidal effect was significantly enhanced by increasing the exposure time of the bacteria on the PPy-GO 2–180# electrode. With the increase of tapping time from 10 min to 25 min, the damage effect of TENG electric field stimulation on bacterial structure became more significant, which was consistent with the results of antibacterial test. Fluorescent dyes such as acridine orange/propidium iodide (AO/PI) were used to stain live/dead cells to investigate the cell viability. It was observed that prolonged finger-tapping of the PPy-GO 2–180# electrode (from 10 to 25 min) further enhanced its bactericidal activity. The results of live/dead staining were consistent with those of OD600 measurements and agar-plate antibacterial-tests. The synergistic effect of electric field stimulation generated by PPy-GO composite TENG is a key factor to enhance the efficacy of bacterial growth inhibition. Electrical stimulation, as a non-traditional antimicrobial means, has demonstrated its potential in the medical field through a variety of mechanisms, especially in promoting wound healing and preventing infection.

#### 5.1.1 Inhibit bacterial growth

Skin wound resistant bacterial infections pose a major health threat, characterized by the persistence of the microbial environment, continuous intensification of oxidative stress, imbalanced immune regulation, and suboptimal state of angiogenesis. Among them, *S. aureus*, as the main gram-positive pathogen, plays a dominant role in skin wound infection (Zhang Y. et al., 2024). Studies have shown that bacterial load is negatively correlated with the rate of wound healing, so reducing bacterial load is a key strategy to reduce the risk of wound infection (Soeselo et al., 2022; Caldwell, 2020).

Ju et al. (2024) innovatively proposed a “win-win co-operation” strategy for photothermal electrical stimulation (PES) combination therapy by designing a MXene-doped wearable composite ionogel patches. The patch utilizes Ti3C2Tx (MXene), a transition metal carbide with a two-dimensional lamellar structure, exhibits good light absorption in the near-infrared region, and combines with opto-electric stimulation to build constructing a robust and ecofriendly composite patch. The doping of MXene in ionogel significantly enhanced the photothermal therapy (PTT) efficacy of the patch. Through the dual action of photothermal effect and electrical stimulation, it effectively inhibited the expression of inflammatory factors, synergically improved the antibacterial effect, and thus accelerated the wound healing process. The results showed that the PES group and the PTS group exhibited significantly better antibacterial activity than the other control groups against *E. coli* (Gram-negative) and *S. aureus* (gram-positive), which was manifested by a significant reduction in the number of bacterial colonies and significant changes in the morphology of bacterial cell membranes. Further validation of the mouse model of total skin defect infection showed that the PES group showed the fastest healing rate after 9 days of treatment.

#### 5.1.2 Enhancing the effect of antibacterial drugs

The dressing system combined with antibacterial drugs shows multiple advantages in wound management, not only providing accurate antibacterial treatment, but also effectively promoting extracellular matrix (ECM) remodeling, enhancing cell activity, and thus accelerating wound healing process. Antibiotics, as the cornerstone of antimicrobial drugs, are directly applied to wound dressings to ensure adequate local bactericidal concentration at the infected site and reduce the occurrence of resistance through controlled release strategies. In addition, inorganic nanoparticles such as silver nanoparticles (AgNPs) and gold nanoparticles (AuNPs) have become new stars in the antibacterial field by interfering with the structure and function of bacterial membranes, resulting in bacterial death. Natural antimicrobials can not be ignored, honey with its antibacterial, anti-inflammatory and antioxidant properties, for wound treatment to provide rich nutritional support; The essential oils showed multi-dimensional biological activities such as antiviral, antioxidant, insecticidal, anticancer and anti-allergic. Herbal extracts, such as alfalfa, gallate, tannic acid and curcumin, have also attracted extensive attention due to their remarkable antibacterial properties. Antimicrobial peptides, as an outstanding representative of bioactive peptides, have opened up a new path for antimicrobial treatment with their broad-spectrum antibacterial activity and strong inhibition ability against gram-positive and Gram-negative bacteria, fungi and even viruses (Gou et al., 2024).

### 5.2 Enhanced cell migration and proliferation

As a biological regulatory instrument, ES has demonstrated a profound impact on cell activity, including the promotion of key processes such as proliferation, growth, migration, and stem cell differentiation. Its effects span both normal and pathological states, involving the regulation of ion transport, gene transcription, protein synthesis, and reactive oxygen generation, as well as the release of chemokines and cytokines. To complex physiological activities such as synaptic extension and cell migration. A large number of studies have shown that bioelectrical stimulation has a significant effect on promoting the regeneration and repair of damaged tissues (Meng SY. et al., 2021). Cell proliferation, as a key process of cell number expansion, is an indispensable link in wound healing, which drives new cells to fill tissue defects in the wound area and lays the cytological basis for wound healing. Especially in the early stages of wound healing, such as within 48 h after wound formation, cell proliferation contributes as much as 73% to wound closure of vascular smooth muscle cells (SMCs), highlighting their central role in the process of “filling the gap” (Ammann et al., 2019). When exploring the mechanism of skin injury repair, the importance of cell migration as a key link to cover and seal the wound is self-evident. In particular, the inward directional movement of keratinocytes from the edge of the wound is the cornerstone of rebuilding the epidermal barrier and restoring its physiological protection function. In this process, the mechanical signal transduction inside the cell works in concert with the external traction force to profoundly regulate the healing process and the tight closure of the wound (Holt et al., 2020).

#### 5.2.1 Migration and proliferation of epithelial cells

In the skin tissue structure, the upper layer is maintained by the interfollicular epidermis and the niches of the hair follicle. These stem cells are not only responsible for the formation of the layered epidermal structure, but also give the skin a protective coat. When the skin is damaged, the damaged epithelial barrier immediately triggers the generation of endogenous electric fields (EFs), which are driven by ionized channels and maintained by intercellular connections. Sensitive epithelial cells are able to pick up these weak electrical field signals and migrate to the wound center as a coordinated whole, maintaining stable relative positions between cells, a process known as epithelial regeneration, which aims to rapidly restore skin barrier function (Sun et al., 2023; Zhao et al., 2022). This phenomenon not only reveals the key role of bioelectric phenomena in wound healing, but also provides a solid biological basis for wound treatment technology based on electrical stimulation.

**FIGURE 8:**
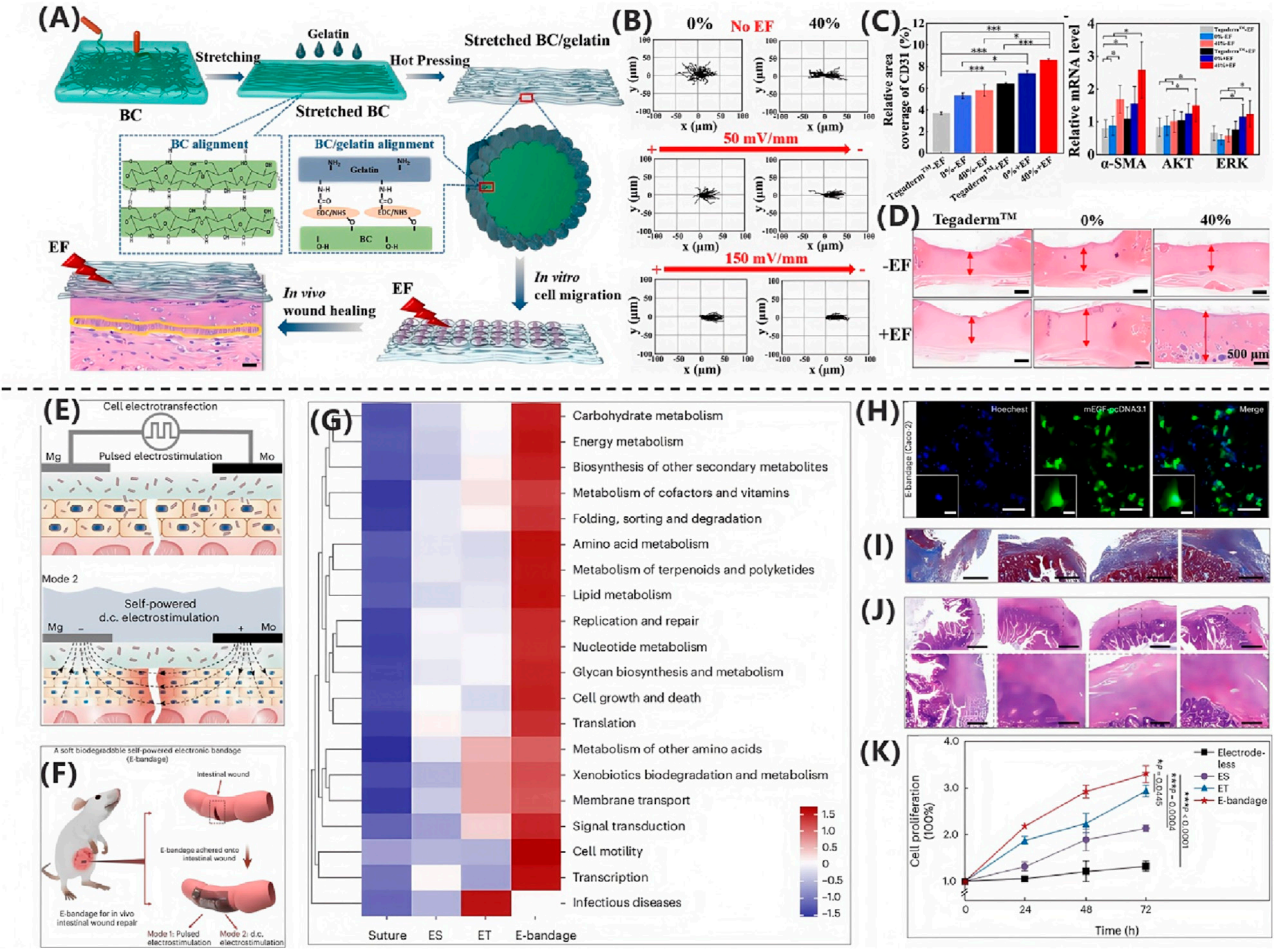
**(A)** Aligned BC/gelatin membranes guided cell migration under EF. **(B)** NIH3T3 showed oriented migration along EF/fibers. **(C)** 40% stretch + EF enhanced CD31^+^ density and α-SMA/AKT/ERK. **(D)** Stretched group improved granulation/vascularization (Wang et al., 2021). Copyright 2024, Springer. **(E)** Pulsed electroporation enabled EGF transfection; Mg/Mo cells generated EF for Ca^2+^-mediated exocytosis. **(F)** e-bandage combined EF + electroporation for intestinal repair. **(G)** e-bandage enriched microbiota metabolism. **(H)** Successful mEGF transfection. **(I)** e-bandage enhanced collagen deposition. **(J)** Improved mucosal healing. **(K)** Dual stimulation (ET + ES) tripled proliferation (Wu et al., 2024). Copyright 2024, Nat Electron.

Figure 8A illustrates the fabrication process of highly aligned bacterial cellulose/gelatin membranes and their application design in wound healing. Based on this material, Figure 8B demonstrates that under an electric field of 150 mV/mm, cells exhibit highly oriented cathode-directed migration trajectories on the 40% stretched membrane, indicating a synergistic guidance effect between electrical stimulation and fiber alignment. Furthermore, Figure 8C reveals that the combined treatment significantly promotes the formation of CD31-positive microvessels and enhances angiogenic capacity. Finally, as shown in Figure 8D, the group treated with the 40% stretched membrane combined with electrical stimulation displays the thickest granulation tissue on day 14, confirming its substantial effect in promoting structural tissue repair (Wang et al., 2021).

The study by Xu et al. (2022) provides an in-depth analysis of the effect of non-contact electrical stimulation (NCES), especially the capacitive coupling electric field (EF), on the migration behavior of human epidermal keratinocyte cell line (HaCaT), a key cell in wound healing. The study shows that NCES can effectively promote cell migration by adjusting the cell arrangement pattern perpendicular to the electric field, which promotes cell expansion along the migration axis, thus accelerating the migration process. To quantify the effect of NCES, the study used scratch experiment as an evaluation tool. The results showed that for HaCaT cells, NCES of 25 and 104 mV·mm^-1^ did not significantly change the healing of scratches, while NCES of 53 and 76 mV·mm^-1^ significantly accelerated the scratch healing process. Further analysis revealed that mitomycin-C-treated HaCaT cells exhibited higher cell density and covered the scratched area mainly through migration mechanism, confirming the significant promotion of HaCaT migration by 53 and 76 mV·mm^-1^ NCES. This finding has important implications for understanding and optimizing the wound healing process. The rapid migration ability of epidermal cells helps to fill the wound defect quickly, and the accelerated migration of upper skin cells is the key to the functional recovery of wound. Therefore, the potential of NCES in promoting skin wound healing cannot be ignored.

#### 5.2.2 Migration and proliferation of fibroblasts

Fibroblasts, with their spindle-shaped to elongated morphological characteristics, exhibit a high degree of plasticity, a property that gives them the ability to participate in a wide variety of cellular and molecular activities. In the complex process of wound healing, fibroblasts actively promote the construction of new connective tissue by proliferating and secreting collagen, and assume the responsibility of deposition and maintenance of collagen and protein networks in the extracellular matrix (ECM). Its role runs through the various stages of hemostasis, inflammation, proliferation and remodeling, and is an indispensable key role in wound healing. In the initial stage, activation of the clotting cascade leads to the formation of fibrin clots, which not only effectively stops bleeding, but also promotes recruitment of immune cells, which in turn triggers the release of pro-inflammatory cytokines and disinfection of the wound site. As the healing process progresses to the later stage of remodeling, the main components of ECM change, and type III collagen is gradually replaced by type I collagen, which is accompanied by the hardening and reinforcement of ECM, and ultimately leads to the formation of mature scars (Parker et al., 2023). Dermal fibroblasts play a central role in the response to skin injury, migrating to the wound area in response to injury signals and depositating basic components there to form the basis of repair together with the granulation tissue generated by the clotting cascade. Fibroblasts run through the whole wound healing cycle, from ECM remodeling to wound contraction, to inflammation regulation and scar formation, which are inseparable from their fine regulation. In particular, fibroblasts exhibit a high degree of plasticity and are able to differentiate from fusiform progenitors into polygonal fibroblasts and myofibroblasts with strong contractile capacity, a process that is carefully regulated by mechanical signals, cytokines and growth factors (especially transforming growth factor-β, TGF-β). Provides potential strategies for achieving scar-free healing (Knoedler et al., 2023).

In the study conducted by Razieh Nazari-Vanani and colleagues, a stretchable TENG was introduced as a stimulant to accelerate cellular proliferation and migration (Nazari-Vanani et al., 2024). Fibroblasts, capable of sensing and responding to electric fields by either approaching or evading them, can be directed in their migratory patterns. To assess the effects, the control and experimental groups were stratified into Group I (no stimulation), Group II (twice daily stimulation), and Group III (three times daily stimulation). Microscopic images depicted the behavior of fibroblasts in the presence and absence of ES over an 8-day period. Initially, on Day 1, cellular counts were equivalent across all groups, with no discernible differences between the control and experimental groups. By Day 5, Group II exhibited a faster proliferation rate compared to the control, and this disparity became more pronounced on Day 8. Furthermore, a scratch assay was conducted on a monolayer of cells to evaluate the impact of TENG stimulation on cellular migration. These micrographs, captured during the interval when cells migrated towards the center of the scratch, comparing the control group to the group receiving ES via TENG. The experiments confirmed that in Groups II and III, ES facilitated a more rapid cellular migration, whereas in Group I, only a minority of cells traversed to the midpoint of the scratch. At 24 h, the relative scratch area in the stimulated groups was 60%, significantly lower than that of the unstimulated group. *In vitro* results indicated that the TENG patch enhanced fibroblast migration by 10% and 20% at 12 and 24 h, respectively, compared to the control group. Collectively, these findings demonstrate that ES effectively promotes fibroblast migration, suggesting considerable potential for TENGs in wound healing applications.

Wu et al. (2024) developed a biodegradable self-powered electronic bandage that accelerates intestinal wound healing through a dual electrical stimulation mechanism. As illustrated in Figure 8E, this device employs pulsed electrical stimulation to induce electroporation in epithelial cells for transfecting EGF-encoding plasmids, while simultaneously utilizing Mg/Mo microelectrode pairs to generate sustained direct current stimulation that promotes EGF exocytosis. Figure 8F demonstrates the application scenario of this electronic bandage on intestinal wounds. *In vitro* experiments confirmed its high transfection efficiency, with Figure 8G showing EGF expression tagged by green fluorescent protein in transfected human intestinal epithelial cells. Animal model validation revealed that dual electrical stimulation significantly enhanced healing factor secretion; Masson’s trichrome and H&E staining in Figures 8H,I demonstrated increased collagen deposition and substantially improved tissue structural integrity in the treatment group. Quantitative data in Figure 8J further confirmed that dual stimulation enhanced cell proliferation rates by 117% compared to single-mode stimulation. Microbial functional clustering analysis in Figure 8K substantiated that this treatment effectively restored intestinal metabolic functions and cellular activity while promoting probiotic microbiota proliferation, providing critical evidence for wound microenvironment remodeling.

#### 5.2.3 Migration and proliferation of vascular endothelial cells

Vascular endothelium, as the innermost barrier of the inner wall of arteries, capillaries and veins, is directly exposed to blood components and cells, which not only finely regulates the dilation and contraction of blood vessels, but also closely regulates the transmembrane transport of solutes, fluids, macromolecules, hormones, platelets and blood cells. It precisely controls local blood flow distribution by regulating vascular tone, and directs inflammatory cells to migrate to damaged or infected areas to initiate repair or defense mechanisms. In addition, vascular endothelium also plays a central role in maintaining blood flow, regulating platelet adhesion and aggregation, leukocyte activation, adhesion and transport, and precisely balancing coagulation and fibrinolysis systems, profoundly affecting the dynamic balance of immune response, inflammation regulation and angiogenesis (Krüger-Genge et al., 2019).

### 5.3 Influence on tissue blood flow

The core of vascularization lies in the mechanism of cell proliferation and directed migration, which provides indispensable nutrition and oxygen support for tissue regeneration and constitutes a crucial link in the wound healing process, directly determining the efficiency of the healing rate (Fu et al., 2023). Specifically, vascular endothelial cells play an important role in the process of angiogenesis and realize the construction and expansion of vascular network through a series of precise regulation. It is worth noting that ES shows its unique repair potential, and significantly upregates the expression levels of VEGF, matrix metalloproteinase 2 (MMP2) and matrix metalloproteinase 9 (MMP9) in the ischemic flap area by effectively alleviating the inflammatory response and apoptosis process triggered by oxidative stress. This regulatory effect further promoted the increase of microvessel density and the significant increase of the number of new vessels, injected strong impetus into the angiogenesis process, and accelerated the process of tissue repair and regeneration (Chen et al., 2024).

VEGF, as the core regulator of physiological angiogenesis in embryonic development, bone construction and reproductive function, plays a crucial role in promoting angiogenesis (Mao et al., 2022).

On this basis, Zhang YX. et al. (2024) innovatively proposed the “cocktail effect” strategy of synergies between electrical simulation and multiple metal ions, and realized the joint intervention of ES and multiple ions by constructing a wearable ionic triboelectric nanogenerator (iTENG) patch. In particular, copper, as a key nutrient for angiogenesis, synergies with iron ions to significantly promote the expression of VEGF, angiopoietin-1 and fibroblast growth factor-2 (FGF2), thereby enhancing the proliferation and migration of endothelial cells and accelerating the reepithelialization process of wound tissue. The accumulation of metal ions in the wound area showed a significant “cocktail effect”, in which endothelial cell adhesion molecule-1 (CD31) was a key evaluation index of angiogenesis. The results of CD31 staining clearly showed a significant advantage in the degree of angiogenesis in the NCs + EP group. The number of CD31 positive cells and the density of microvessels were much higher than those in other controls, thus accelerating the process of wound healing.

**FIGURE 9:**
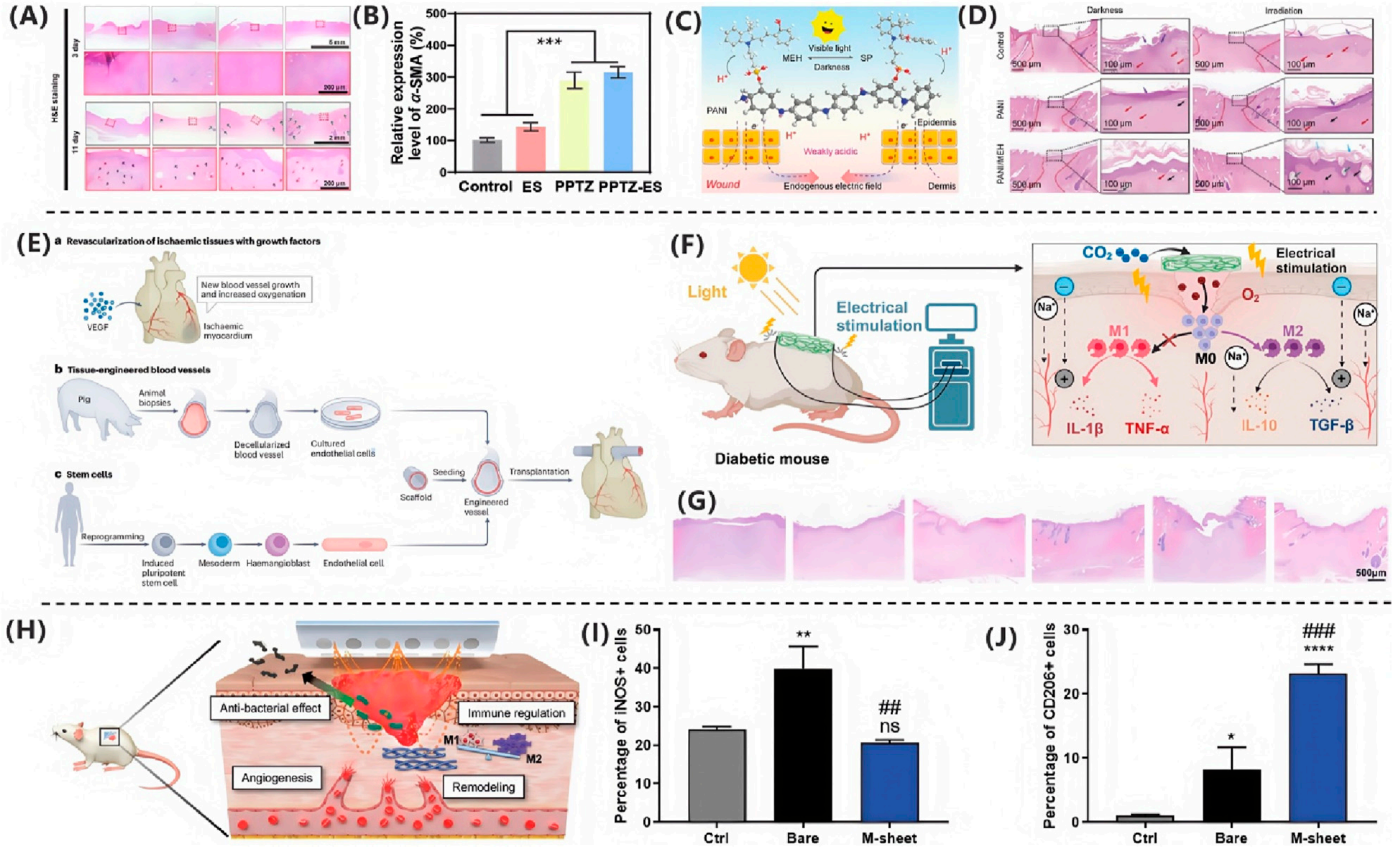
**(A)** PPTZ-ES group achieved complete regeneration with new follicles/vessels. **(B)** α-SMA increased 304.49%, confirming vascular maturation (Zheng et al., 2024). Copyright 2024, Nano Res. **(C)** MEH generated photocurrent (60 μA·cm^-2^) and antibacterial acidity (pH ≈ 3.7). **(D)** Phototherapy enabled 81% closure by day 7 (Yin et al., 2023). Copyright 2023, Adv Healthc Mater. **(E)** ES upregulated VEGF-A via PI3K/AKT, reducing inflammation (Pérez-Gutiérrez and Ferrara, 2023). Copyright 2023, Nat Rev Mol Cell Biol. **(F)** PVNP-SP hydrogel + ES increased M2 (CD206^+^) and decreased M1 markers. **(G)** SepMN enhanced closure via antibacterial/M2 effects (Liu et al., 2024). Copyright 2024, Chem Eng J. **(H–J)** M-sheet’s redox field inhibited bacteria, promoted M2 polarization, and accelerated healing (Kim JU. et al., 2024). Copyright 2024, Adv Healthc Mater.

Zheng et al. (2024) demonstrated that multifunctional PPTZ hydrogel combined with electrical stimulation significantly promoted tissue regeneration and healing in diabetic wounds. Key evidence for tissue blood flow regulation was observed in Figure 9A, which revealed dense neovascularization indicated by red arrows. Figure 9B quantitatively confirmed via α-SMA immunofluorescence that vascular smooth muscle cell expression reached 304.49%, substantiating that the hydrogel synergistically with electrical stimulation enhanced neovascularization and improved local microcirculation.

Yin et al. (2023) developed a polyaniline-based conductive antibacterial hydrogel PSP for treating infected chronic wounds. Animal experiments confirmed its significant therapeutic efficacy. As shown in Figure 9C, complete wound healing was achieved after 14 days of treatment combining PSP hydrogel with electrical stimulation. Histological analysis further revealed that compared to electrical stimulation alone or conventional dressing groups, this treatment group exhibited a 28% significant increase in re-epithelialization extent, a 100% elevation in collagen deposition, and markedly enhanced neovascularization. This superior efficacy stems from the multifunctional synergy of the PSP hydrogel. Fabricated via *in situ* polymerization embedding aniline within a polyacrylamide network and stabilized with sulfonated hyaluronic acid as a macromolecular dopant for polyaniline’s conductive state, its preparation process is detailed in Figure 9D. The resulting PSP hydrogel demonstrates skin-comparable conductivity of 1.05 mS/cm. Its antibacterial mechanism relies on polyaniline’s specific binding to Gram-positive bacterial lipoteichoic acid, thereby disrupting cell wall structural integrity.

### 5.4 Improve the healing rate of chronic wounds

Chronic wounds, defined as wounds that do not show signs of healing for more than 1 month, are characterized by persistent inflammation, a significant delay in the healing process, and complications associated with a high risk of tissue damage and infection. This type of wound covers a wide range of diabetic foot ulcers, pressure ulcers, venous ulcers and radiation ulcers and other common clinical types. In the pathophysiological process of chronic wounds, the imbalance of inflammatory cytokines, abnormal expression of growth factors and changes in proteolytic enzyme activity jointly lead to the difficulty of wound healing or significant prolongation of healing time. Meanwhile, the treatment process may also induce adverse complications such as infection, which brings great challenges to current medical interventions. Innovative treatment strategies are urgently needed (Li and Zheng, 2024).

VEGF plays a pivotal role in wound healing by regulating angiogenesis and inflammatory responses. Studies confirm that pathological VEGF upregulation leads to aberrant vascular leakage and inflammatory cell infiltration, creating a fibrotic microenvironment that impedes the healing process. As shown in Figure 9E, targeted VEGF therapy, achieved through delivering exogenous VEGF or utilizing tissue-engineered vascular grafts, significantly promotes functional neovascularization in ischemic tissues. This improves local perfusion and oxygenation, accelerating re-epithelialization. Furthermore, research elucidates that stem cell therapy synergistically enhances vascular network reconstruction by inducing the differentiation of pluripotent stem cells into endothelial cells and promoting the secretion of factors like VEGF. This effectively reduces the healing duration of chronic wounds through mechanisms involving suppression of excessive inflammatory responses and mitigation of scar formation (Pérez-Gutiérrez and Ferrara, 2023).

### 5.5 Impact on scars

Fibroblasts play a central role in the homeostasis of the ECM, responsible for the synthesis, secretion and remodeling of key components of the ECM, especially collagen and elastin, the two most abundant structural proteins. Collagen, as the cornerstone of skin ECM, gives it excellent elasticity and strength. Of its various types, type I collagen dominates adult skin, but after trauma, its place is temporarily replaced by type III collagen, which then undergoes remodeling to restore homeostasis. Elastin, on the other hand, is synthesized during wound healing and, although it accounts for only a small fraction of ECM (0.6%–7.9%), is essential for reducing scar contraction, promoting skin regeneration and giving the wound area the necessary flexibility. Reconstruction of the elastic fiber network is essential for full recovery of skin function. However, inadequate elastin regeneration, accompanied by excessive accumulation of collagen synthesis, often leads to an undesirable healing outcome-the formation of scars that are significantly less flexible and elastic than normal dermis. When skin is damaged, ECM proteins undergo a series of finely regulated repair processes aimed at restoring skin integrity, structural stability, and internal environmental balance. This process begins in the hemostatic phase, during which extracvascular collagen (mainly type I) activates platelets, interacts with ECM proteins (collagen, fibronectin, von Willebrand factor, etc.) through platelet receptors (e.g., glycoprotein VI), releases soluble mediators such as cyclic AMP, and induces the release of blood cells. Promote platelet adhesion and release of growth factors and adhesion glycoproteins. Following the inflammatory phase, PDGF (platelet-derived growth factor) is activated to recruit fibroblasts to the wound site, prompting them to synthesize collagen and glycosaminoglycan, providing favorable conditions for cell migration. In the growth stage, the temporary fibrin matrix is gradually replaced by a new matrix composed of collagen fibers, proteoglycans and fibronectin. During this process, fibroblasts are stimulated to replace the original fibrin clots with hyaluronic acid, proteoglycan and fibronectin to promote the formation of mature collagen fibril. As the scar matures, type III collagen is gradually replaced by type I collagen, which enhances the tensile strength of the scar. In order to maintain skin elasticity, the reconstruction of elastin in ECM is particularly critical. It is involved in cell migration, matrix construction, protease production, tension regulation and defense response, and actively contributes to wound healing and maintenance of skin homeostasis by enhancing cell adhesion, diffusion, proliferation and signal transduction mechanisms (Gardeazabal and Izeta, 2024; Gajbhiye and Wairkar, 2022).

In the pioneering research of Xiang et al. (2023) successfully generated rectangular pulse single-phase microsecond pulse electric filed (μsPEF) waveforms using their self-developed solid-state Marx generator (SSMG). This wave shape exhibits a rapid rising/falling edge in the nanosecond range to stimulate cells, thereby inducing a microsecond pulsed electric field mediated wound healing mechanism dependent on actin and collagen. It was found that a short duration of 750 V/cm μsPEF induced rapid migration of fibroblasts over a long period of time. In addition, the study demonstrated that μsPEF stimulation can effectively regulate the recombination of actin cytoskeleton and the conversion of focal adhesion, thus significantly promoting the migration rate of fibroblasts in a two-dimensional cell culture system. This accelerated fibroblast migration phenomenon is accompanied by co-alignment with the ECM in a three-dimensional microenvironment, which together drives the final process of wound healing. To investigate the effects of μsPEF stimulation on the secretion of collagen α2 (COL1A2) or FGF-2 from fibroblasts, we used an enzyme-linked immunosorbent assay (ELISA) to measure the levels of these two molecules in the cell supernatant 48 h after stimulation. The results showed that under the stimulation of 750 V/cm and 1500 V/cm μsPEF, the secretion of COL1A2 increased by 137% and 233%, respectively, compared with the control group, indicating that μsPEF can promote the synthesis of new ECM in fibroblasts. Provide the necessary support for other cells associated with effective wound healing. At the same time, Masson staining results of skin tissue slices also showed that the collagen fiber tissues in the 750 V/cm μsPEF group were well arranged, which was consistent with the results of *in vitro* experiments, and this change was consistent with the characteristics of the remodeling stage of wound healing. Further, to investigate whether μsPEF stimulation leads to collagen degradation, the researchers quantified changes in collagen concentration after electrical stimulation. The results of sodium dodecyl sulfate polyacrylamide gel electrophoresis (SDS-PAGE) showed that, at the same initial concentration, the final concentration of collagen gel decreased with the increase of the μsPEF stimulating electric field intensity, which meant that the degradation of collagen gel occurred to different degrees under the stimulation of different μsPEF intensity. In addition, the collagen gel stimulated by μsPEF was significantly twice as hard as that in the unstimulated group. More importantly, μsPEF effectively promoted the closure of skin wounds *in vivo*, and the closure efficiency along the electric field line was higher. This effect is attributed to faster migration of fibroblasts and better arrangement of collagen fibers. In the end, healing efficiency was significantly improved 2.5 times in the μsPEF stimulated group compared to the unstimulated group. This orderly arrangement of collagen fibers helps to form stable scar tissue, which speeds up the wound healing process.

#### 5.5.1 Promote collagen production

Electrical stimulation, as a means of biological regulation, can significantly enhance the transcriptional activity of PDGF gene, and promote the synthesis and release of PDGF protein. PDGF then activates the proliferation potential of fibroblasts through its specific receptor-mediated signaling pathway and induces their differentiation into myofibroblasts. During this differentiation, the expression level of α-smooth muscle actin (α-SMA), a key marker, was significantly upregulated, marking the transformation of the functional characteristics of fibroblasts. Subsequently, myofibroblasts act synergistically with incomplete differentiated fibroblasts to accelerate the production and deposition of type I collagen in the coarse fiber bundles. In the proliferative stage of wound healing, type I collagen, as the core structural component, constructs the matrix framework supporting cell migration and proliferation. This framework not only provides necessary attachment points and migration paths for all kinds of repaired cells, but also promotes the recovery of tensile strength of repaired tissues in the mature stage, usually reaching between 80% and 85% of the mechanical properties of normal tissues, which is crucial for maintaining the quality of wound repair and functional recovery (Urabe et al., 2024).

Among the many cytokines and growth factors that regulate wound healing, TGF-β has attracted much attention for its far-reaching influence on collagen formation and extensive functions, especially the TGF-β1 subtype, as the core regulatory factor, plays a crucial role in the cellular response at various stages of wound healing. In the proliferative phase, TGF-β1 significantly promoted the proliferation of fibroblasts and the synthesis of ECM, accelerated the construction of meat bud tissue and wound contraction process. Entering the remodeling phase, TGF-β1 ensures a delicate balance between ECM synthesis and degradation by finely regulating the expression of matrix metalloproteinases (MMPs) and their inhibitors, such as TIMP-1. In this process, TGFβR1-mediated Smad2/Smad3 phosphorylation, and then form an active transcription complex with Smad4, deeply regulate the transcription activity of related target genes in the nucleus, dynamically adjust the TGF-β signaling pathway, and enhance the activity of smad2 in the proliferation phase. Promote the accumulation of collagen and the synthesis of type III fibrillar fibers, thus accelerating the regeneration and healing process of wounds. Therefore, the fine regulation of TGF-β1 pathway has a decisive influence on the quality of wound healing (Li LS. et al., 2023).

#### 5.5.2 Inhibition of scar formation

After tissue damage, a series of cellular responses are triggered, in which platelets, endothelial cells, and inflammatory cells work together to induce fibroblasts to initiate ECM deposition. However, uncontrolled secretion of ECM proteins such as collagen can lead to excessive scarring and fibrosis, which can impair the normal structure and function of tissues. Fibrous collagen is the core component of scar tissue, and its abnormal accumulation can lead to the development of pathological scars, which is not only a threat to life and health, but also a major factor in the decline of life quality and the increase of economic burden (Raote et al., 2024). In this context, the concept of scarless healing emerged, which represents an ideal state of tissue repair aimed at maximizing the restoration of tissue structure and function while preserving the original beauty and elasticity of the skin. Therefore, effective inhibition strategies for scar formation are particularly important and have far-reaching significance for improving wound healing quality and patient prognosis.

Fibrosis, as a pathological process, refers specifically to the abnormal deposition of the ECM rich in collagen I (Col1), which is a response to the infiltration of immune cells and the onset of symptoms after tissue injury. In areas of tissue damage, ECM deposits initially provide essential structural support. However, when opal secretion is out of control, ECM accumulates excessively and exceeds tissue repair needs, resulting in the formation of fibrotic scars. Reducing the generation of fibrotic scars, especially by interfering with the proliferation of fibrotic cells, has significant significance in alleviating motor dysfunction (Dorrier et al., 2021).

### 5.6 Influence on the inflammatory stage

In the complex physiological process of wound healing, moderate inflammatory response plays an indispensable role, which effectively clears the necrotic tissue and resists the invasion of exogenous pathogens, and is a key link in the healing sequence. However, chronic wounds are often trapped in a continuous inflammatory cycle due to adverse factors such as abnormal differentiation of macrophages, imbalance of neutrophil metabolism, excessive release of pro-inflammatory cytokines and bacterial infection. This long-term inflammatory state is extremely harmful, not only hindering the wound healing process, but also promoting the formation of excessive scar tissue. In view of this, regulating and reducing the level of excessive inflammatory response during wound healing has become an extremely challenging task to accelerate chronic wound healing. In this context, the innovative strategy of integrating electrical stimulation technology with conductive dressing platform has shown significant advantages, which can accurately regulate the expression levels of growth factors and inflammatory factors, and effectively prevent excessive extension of inflammatory period. Furthermore, anti-inflammatory therapy, as an adjunct, plays a crucial role in facilitating the smooth transition of the wound from the inflammatory phase to the proliferative phase. This transformation is a crucial link in achieving full wound healing and functional recovery (Xu et al., 2021). In summary, it is expected to open up a new path for efficient healing of chronic wounds through the comprehensive application of multiple strategies such as electrical stimulation, conductive dressing and anti-inflammatory therapy.

#### 5.6.1 Remission of inflammation

Inflammation, as an inherent stage in the natural process of wound healing, aims to clear the wound of cellular debris and latent pathogens, ensuring the purification of the healing environment. However, when the process of infiltration or clearance of the damaged tissue by white blood cells is blocked, or it is not effectively treated by apoptosis mechanism, it will prevent the smooth transition of the wound to the proliferation stage, and then delay or inhibit the healing process. At the micro level, persistent inflammatory response is one of the core mechanisms of chronic wound formation and has a profound impact on the development of wound healing disorders (Holzer-Geissler et al., 2022).

In a pioneering study by Du et al. (2021), TENGs patch was designed to integrate an Mg-Al layered double hydroxides (LDH) functionalized carrier surface engineered electrode designed to synchronise ES with precision drug loading/release control to accelerate the healing process of infected wounds. The study results showed that on the 10th day of treatment, H&E staining analysis showed that the MLDH@Al film (MSETENG) treatment group showed complete epidermis with significant reduction of inflammatory cells, and the generation of new hair follicles was observed, and the boundary between regenerated tissue and host tissue was blurred, showing a good healing trend. In contrast, the control and Mg-Al LDH nanosheets on Al foils (LDH@Al) groups showed epidermal imperfections, significantly increased dermal defects, and significant concentrations of inflammatory cells (marked by green circles), highlighting the superior effect of electrical stimulation in reducing the inflammatory response and shortening the inflammatory period. This finding not only validates the potential of the TENG patch in promoting rapid healing of infected wounds, but also provides strong support for the development of future treatment strategies for chronic wounds.

#### 5.6.2 Reduce the release of inflammatory mediators

In the complex mechanism of wound healing, inflammatory mediators (including prostaglandins, cytokines, proteases, reactive oxygen species and nitrogen species) are not only the key trigger for the initiation of inflammatory response, but also the indispensable regulator of the healing process. In particular, during inflammation, the number of subepithelial myoblasts surges, and cytokines such as IL-17 directly stimulate these cells to produce a variety of mediators including IL-6, IL-8, and MCP-1, further exacerbating the inflammatory response. The focus is on TNF and IL-6, two core cell agents that play a critical role in both the initiation and resolution of inflammation. By inducing the expression of Ptgs2 gene, TNF promotes the production of COX-2 and then increases the PG level, which aggravates the formation of vascular permeability, leukocyte infiltration and edema in the early stage of acute inflammation. IL-6, on the other hand, relies on its homologous receptor IL6Ra to activate the JAK/STAT signaling pathway on immune cells, thereby driving the cascade amplification of inflammatory response (Crifo and MacNaughton, 2022).

The study of Ferreira et al. (2021) focused on the specific role of ES in the early healing process of palatine mucosa in Swiss mice. By comparing the control group with the electrical stimulation group, it was found that the concentrations of inflammatory biomarkers such as IL-6, TNF-α and IL-1β were significantly reduced in the ES group at multiple time points (day 1, 3 and 5), especially in the regulation of TNF-α and IL-1b, effectively interrupted the positive feedback cycle of inflammation. In addition, although VEGF concentrations decreased on day 5, group ES exhibited better anti-inflammatory properties overall, helping to accelerate the wound healing process. These findings not only deepen our understanding of the anti-inflammatory mechanism of ES in wound healing, but also provide strong support for future clinical treatment strategy optimization.

Liu et al. (2024) demonstrated that PVNP-SP hydrogel combined with photoelectric stimulation significantly accelerated diabetic wound healing through a dual mechanism. Figure 9F illustrates this therapeutic strategy effectively modulated macrophage polarization: immunofluorescence quantification confirmed a marked reduction in the proportion of iNOS^+^ M1 macrophages and a concurrent increase in CD206^+^ M2 macrophages within the wound. ELISA assays revealed decreased levels of pro-inflammatory cytokines TNF-α and IL-1β alongside elevated levels of anti-inflammatory cytokines IL-10 and TGF-β, indicating regulated inflammatory phase resolution. Histological analysis in Figure 9G further demonstrated accelerated healing progression: H&E staining showed significantly reduced inflammatory cell infiltration and accelerated epidermal re-epithelialization in the treatment group by day 7. By day 10, complete neo-epidermis formation was observed with thickened granulation tissue and reduced dermal gaps. Masson’s trichrome staining confirmed substantially enhanced collagen deposition and organization.

#### 5.6.3 Polarization of immune cells

Precisely regulating the behavior of immune cell subpopulations has become a core strategy in the field of wound healing therapy, where electric field technology shows significant therapeutic potential by influencing the polarization state of immune cells, especially macrophages. As the main carrier of ES, electroactive polymer materials directly act on immune cells through electrical signals at the cell-material interface to regulate their polarization, migration, differentiation, phagocytosis and other biological activities. Macrophages play a dual role in wound healing: the M1 phenotype dominates the pro-inflammatory response, while the M2 phenotype promotes anti-inflammatory and repair processes. As an effective means to regulate the polarization of macrophages, electric field technology can flexibly induce their transformation into M1 or M2 phenotypes, which is crucial for optimizing the transition from the inflammatory phase to the remodeling phase of wounds. In particular, enhancement of M2 polarization is seen as a key driver of anti-inflammatory effects during wound recovery (Barman and Jhunjhunwala, 2023).

Gu et al. (2022) conducted an in-depth study on the fine regulation of ES on the classically activated (M1)/alternatively activated (M2) polarization balance of macrophages. We evaluated the effect of different ES intensities on the cytokine mediated M1/M2 polarization span using lipopolysaccharide (LPS)/IFN-γ and IL-4 induced mouse models of bone marrow‐derived macrophages (BMDMs). In the culture system maintaining the equilibrium state of BMDMs in M1/M2, by applying ES with a specific intensity, it was found that although the degree of polarization of macrophages increased with the increase of ES intensity, too high intensity was not conducive to the optimal induction of polarization. The 500 mV voltage was identified as the optimal ES condition. Further analysis showed that M1-polarized macrophages showed strong pro-inflammatory properties, while M2-polarized macrophages had high phagocytosis and scavenging ability and nutrient synthesis ability, thus promoting the process of tissue repair. Therefore, ES, as an effective tool to regulate the polarization state of macrophages, provides a new target and strategy for precise dry-pretissue repair and wound healing process.

The M-sheet developed by Kim JU. et al. (2024) as depicted in Figure 9H, significantly accelerates diabetic wound healing by producing endogenous electrical stimulation through spontaneous redox reactions. Its electrical stimulation properties enhance the migration and proliferation of keratinocytes and fibroblasts, thereby expediting wound closure. Key mechanisms include modulation of inflammatory responses: Figures 9I,J demonstrate that the M-sheet induces macrophage polarization toward the M2 phenotype, markedly reducing pro-inflammatory factor iNOS expression while increasing anti-inflammatory marker CD206, effectively ameliorating chronic wound inflammation. Concurrently, the material enhances angiogenesis and tissue remodeling by promoting vascular endothelial growth factor expression and facilitating organized collagen deposition. *In vivo* experiments confirm that the M-sheet substantially reduces the wound healing period in diabetic mice, improves re-epithelialization rates, and enhances tissue functionality, offering an innovative battery-free electrical stimulation strategy for chronic wound management.

### 5.7 The impact of individual differences on electrical stimulation treatment outcomes

The individual factors of patients influence the efficacy of electrical stimulation. In the study by Zanto et al. (2021), the researchers pointed out that individual differences have a significant impact on the therapeutic effects of transcranial alternating current stimulation (tACS), primarily manifested in two aspects: neuroanatomical structure and neurophysiological characteristics. From a neuroanatomical perspective, variations in skull thickness, cerebrospinal fluid distribution, subcutaneous fat content, gyral morphology, and local tissue heterogeneity among individuals lead to differences in electrical conduction impedance. As a result, the same intensity of tACS can produce significantly varying electric field strengths in the cerebral cortex of different individuals. From a neurophysiological standpoint, due to inherent differences in endogenous neural oscillatory activity, the intervention effect of tACS is most pronounced when the stimulation frequency is close to the individual’s intrinsic peak oscillation frequency. Research data indicate that in long-term theta-band stimulation experiments, subjects whose baseline theta peak frequency was closer to the 6 Hz stimulation frequency exhibited more significant improvements in multitasking ability after the intervention. Further analysis revealed that the synergistic effect of simulated electric field strength and baseline theta peak frequency, these two key factors, could explain 54%–65% of the interindividual variability in tACS intervention outcomes. This finding underscores the importance of considering individual differences in the clinical application and experimental design of tACS.

In the study by Mahmood et al. (2019), ES was proven to effectively improve impaired wound healing in diabetic animals. Using an alloxan-induced diabetic rabbit model, the results showed that diabetic animals receiving ES treatment (T3 group) exhibited a significant acceleration in wound healing, with a reduction in wound area of 0.3–0.2 cm^2^ per treatment session. Although their healing rate was slower than that of non-diabetic animals (T2 group), it was significantly better than that of the diabetic control group receiving only insulin treatment (T4 group). Notably, the T4 group failed to achieve complete wound healing after seven treatment sessions, whereas the T3 group reached full healing within the same timeframe. This result clearly demonstrates the specific therapeutic advantage of ES for diabetic wounds. Mechanistically, ES may promote diabetic wound repair through multiple pathways: first, by simulating endogenous injury electric fields to directionally guide the migration of epithelial cells and fibroblasts; second, by activating the MAP kinase signaling pathway to enhance cell proliferation and improve the recruitment of inflammatory cells (neutrophils and macrophages); additionally, ES exhibits significant metabolic regulatory effects, including lowering blood glucose levels (by enhancing insulin receptor sensitivity and glycogen synthesis), reducing oxidative stress (through the neutralization of free radicals by exogenous electrons), and improving lipid profiles (manifested as a mild increase in HDL and reductions in LDL and triglyceride levels). These findings not only provide a new therapeutic strategy for impaired wound healing in diabetes but also offer important insights into the biological effects of ES.

In the study by Khalil and Merhi (2000), the researchers explored the effects of non-invasive transcutaneous electrical nerve stimulation (TENS) on sensory nerve activation in aged rats and its impact on wound healing. With aging, the regulatory capacity of sensory nerves on cutaneous vascular responses declines, leading to weakened neurogenic inflammation and thereby impairing wound repair efficiency. The experiments found that low-frequency (5 Hz) TENS stimulation significantly enhanced blood flow responses in the hind paw pads of aged rats, with vasodilatory effects similar to those observed in young rats, indicating that the sensory nerves of aged individuals retain responsiveness to low-frequency electrical stimulation. In a thermal injury model, aged rats receiving TENS treatment showed significantly shorter wound healing times (14.7 ± 0.2 days) compared to the sham-treated group (21.8 ± 0.3 days), confirming that low-frequency TENS can promote tissue repair by activating peripheral sensory nerves. Furthermore, the study found that the sympathetic nervous system plays a limited role in the vascular responses induced by low-frequency TENS, as no significant regulatory effect of sympathetic nerves on vasodilation was observed in either young rats with sympathetic blockade by phentolamine and guanethidine or aged rats under low-frequency stimulation conditions.

## 6 Conclusion

ES demonstrates significant therapeutic potential as a non-invasive modality for wound healing, as evidenced by recent research. This article systematically explores ES’s scientific foundations, encompassing its mechanisms of action (MOA), application technologies, and MS criteria. Detailed analysis addresses ES’s medical applications while evaluating its clinical value. Although progress has been achieved in ES-mediated healing research, persistent challenges require further resolution.

### 6.1 Accuracy

The cellular microenvironment in wound regions exhibits extreme complexity, encompassing diverse cytokines, GFs, and intricate intercellular interactions. This complexity results in marked variations in ES responsiveness across wound types, as documented in reference. Furthermore, interpatient heterogeneity in local tissue physiology-including circulatory status and neural functionality-may lead to differential responses to identical ES parameters.

ES-mediated effects on wound microenvironments may extend beyond localized areas, potentially inducing systemic physiological alterations. For instance, ES may exert systemic effects through modulating systemic inflammatory responses, immune regulation (IR), or metabolic processes, thereby indirectly influencing patients’ global health status (Gupta and Krasnoslobodtsev, 2024). However, the mechanisms and scope of ES-induced systemic effects-including their spatial distribution and intensity gradients-remain poorly characterized, necessitating further mechanistic investigations. The ES dose-response relationship constitutes a critical research focus. Variations in ES parameters-including current intensity, frequency, waveform, and stimulation duration (SD)-demonstrate differential healing impacts. Establishing optimal ES parameters and their patient-specific adaptation based on wound types and individual variations is paramount for achieving personalized ES therapy (PET) and treatment optimization. Future studies must investigate ES parameter-therapeutic efficacy correlations while developing protocols to optimize ES regimens according to wound characteristics and patient physiology.

### 6.2 Integration

Integrative therapeutic strategies significantly enhance wound healing outcomes. ES combined with drug therapy (DT) demonstrates dual benefits: enhancing pharmacological efficacy while reducing drug resistance development. This synergistic effect potentially stems from ES-induced modulation of cell membrane permeability (CMP) that facilitates intracellular drug transport, coupled with enhanced drug action through cellular signaling pathway regulation (Zhao et al., 2020; Ryu et al., 2024). Furthermore, ES-biomaterial integration improves biomaterial performance via controlled release (CR) of bioactive substances and enhanced host tissue integration, collectively elevating healing quality (Ren et al., 2024).

Current research on ES-based combination therapy (CT) remains in the exploratory phase. While existing studies suggest potential synergistic effects between ES and adjunct therapies, their mechanistic foundations (MFs) and optimal implementation protocols (OIPs) require systematic validation. Future investigations must elucidate ES-therapeutic modality interactions and develop CT optimization frameworks to maximize therapeutic outcomes. This necessitates establishing ES parameter optimization matrices, selecting drug therapy/biomaterial components through compatibility screening, and conducting rigorous safety-efficacy (SE) assessments of CT regimens. Furthermore, researchers must systematically investigate AEs and RPs associated with ES-based CT to ensure TS. This safety-centric approach will enable development of precision-engineered CT regimens that optimize ES parameters while mitigating MRs, ultimately providing novel therapeutic strategies for CWs and HHWs.

### 6.3 Implantable devices

Traditional implantable medical devices (such as pacemakers) face two core challenges: limited energy supply and insufficient biocompatibility. Currently, such devices primarily rely on disposable batteries or external power sources, which not only have limited endurance but also require secondary surgeries to replace batteries, increasing patients’ medical risks and financial burdens. Additionally, the large size and rigid nature of traditional batteries restrict the miniaturization and flexibility of these devices, while the suboptimal biocompatibility of some materials can trigger immune rejection, chronic inflammation, and tissue fibrosis, thereby affecting device performance and long-term safety.

To overcome these limitations, self-powered technologies have become a research hotspot, among which TENG and PENG show significant potential. These technologies capture mechanical energy from human motion, heartbeat, or respiration and convert it into electrical energy, enabling self-powering of devices. For example, a TENG-based symbiotic pacemaker can directly drive pacing circuits using the mechanical energy of heartbeats, significantly reducing reliance on traditional batteries. Meanwhile, supercapacitors, with their high biocompatibility, rapid charge-discharge characteristics, and ultra-long cycle life, are considered ideal energy storage units, further enhancing system sustainability.

In terms of material design, the development of new electrodes and encapsulation materials has become crucial. Flexible electrodes (such as organic semiconductors, CPs, or hydrogel-based materials) mimic the mechanical properties of biological tissues, significantly reducing the modulus mismatch at the implantation interface and minimizing tissue damage risks. Hydrogel electrodes can also optimize conductivity by incorporating conductive nanomaterials (such as carbon nanotubes or graphene) while maintaining excellent biocompatibility. Encapsulation materials must balance biostability and degradability—for instance, flexible polymer materials like poly(lactic-co-glycolic acid) (PLGA) can protect electronic components while safely degrading after service, eliminating the need for secondary removal surgeries.

However, several technical bottlenecks remain in this field: First, the long-term stability and energy conversion efficiency of TENG/PENG in dynamic physiological environments require further optimization. Second, electrode materials must balance conductivity, flexibility, and biocompatibility, and the long-term interaction mechanisms at the tissue interface still need in-depth exploration. Additionally, systematic research on the degradation rate control of biodegradable materials and their long-term effects on the body is still lacking.

Looking ahead, with the integration of multidisciplinary fields (such as materials science, micro/nano energy, and artificial intelligence), implantable devices will evolve toward intelligence and personalization. By incorporating IoT and remote monitoring technologies, real-time data transmission and adaptive control become possible, driving innovation in precision medicine. Breakthroughs in this field will not only address existing clinical pain points but also expand into emerging applications such as neuromodulation and wearable medical technologies (Cui et al., 2024).

### 6.4 Self-powered

Conventional ES regimens require patients to connect to external power sources (EPSs), which restricts mobility and elevates infection potential (IP) due to wire penetration through wound dressings (WDs) or direct skin contact. To address these limitations, developing self-powered ES devices has become a research priority. Current SPD variants primarily include biofuel cells (BFCs), triboelectric nanogenerators, and piezoelectric materials (PEMs) (Gupta and Krasnoslobodtsev, 2024). These systems enable personalized, continuous energy delivery (CED) while reducing EPS dependence and improving patient mobility (PM).

SPDs face critical technical challenges (CTCs) including limited reactant supply (RS), environmental sensitivity, and suboptimal energy conversion efficiency (ECE) (Preetam et al., 2024). These limitations compromise SPD reliability and practical applicability (PA). Addressing these CTCs requires interdisciplinary integration of materials science (MS), energy technology, and bioengineering to develop next-generation SPDs with enhanced stability, user-friendliness, and biosafety.

### 6.5 Clinical translation

Current clinical practice lacks universally accepted macroscopic assessment criteria for WHP. Specialized evaluation systems (SESs) remain underdeveloped for specific wound types (SWTs) including burn wounds and diabetic foot ulcers (DFUs), complicating therapeutic efficacy comparison and analysis. While tissue biopsy techniques enable direct observation of cell proliferation (CPx), differentiation, and extracellular matrix (ECM) synthesis, inconsistent operational protocols (OPs) for sampling locations (SLs), frequency, and analytical methods (AMs) create protocol variability (PV). This PV compromises data accuracy (DAc) and comparability of critical parameters like CPx, ultimately hindering definitive TEE of ES interventions.

To enhance the accuracy and reliability of WHP assessment, future studies must develop and validate standardized assessment tools and methods (SATMs). This includes defining optimal SLs, frequency, and AMs for tissue biopsies to ensure data consistency (DCn) and comparability. Additionally, researchers should integrate non-invasive evaluation techniques such as imaging modalities and biomarker analysis to provide novel perspectives for WHP monitoring. These advancements aim to achieve precise and objective evaluation of the WHP, thereby establishing a robust scientific foundation for therapeutic TEE of interventions like ES. Such progress is critical for optimizing treatment protocols (TOs), improving TOs, and ultimately enhancing patient prognoses.

### 6.6 Clinical selection strategy

In the treatment of chronic wounds, the selection of minimally invasive implantable power sources requires comprehensive consideration of energy requirements, biocompatibility, safety, and clinical applicability. For short-term treatment needs, biodegradable primary batteries or supercapacitors are ideal choices due to their no-need-for-removal and minimally invasive characteristics, but their degradation mechanisms and energy density need optimization. For long-term treatment, rechargeable secondary batteries or human energy harvesting technologies (such as nanogenerators and biofuel cells) show greater potential, though challenges like charging convenience, output stability, and biocompatibility must be addressed. Wireless energy transmission technologies (such as near-field wireless charging, ultrasound, or photovoltaic power) can reduce infection risks associated with physical connections, but their efficiency, safety, and device size still require improvement. Clinical decisions should be based on wound type, treatment duration, and patient-specific factors, prioritizing flexible, lightweight power solutions with high tissue compatibility, while integrating dynamic energy management and multifunctional technologies to achieve efficient and safe chronic wound healing. In the future, through material innovation, structural optimization, and intelligent power management, minimally invasive implantable power sources are expected to play a greater role in precision medicine (Xu M. et al., 2024).

Non-invasive Degradative Wave (DW) electrical stimulation has been shown to effectively enhance blood flow and hemoglobin levels in acute skin wounds without affecting wound closure time, indicating its potential application in promoting acute wound healing. Although ES has proven beneficial for wound repair, the mechanisms of DW, optimal device selection, and dosing protocols require further exploration. Using a temporal skin biopsy model combined with non-invasive assessment methods such as spectrophotometric intracutaneous analysis (SIAscopy) and full-field laser perfusion imaging (FLPI), studies have found that DW significantly improves blood flow perfusion and oxygenation in acute wounds. However, due to limitations such as small sample size (n = 20) and insufficient monitoring time points, only more pronounced effect differences could be detected. Future research should expand sample sizes, extend observation periods, and further investigate the role of DW in reducing pathological scars (such as hypertrophic scars and keloids) to facilitate its clinical translation (Ud‐Din et al., 2012).

### 6.7 Comparative efficacy of electrical stimulation versus traditional therapy

Electrical stimulation dressings demonstrate significantly superior antibacterial effects in infected wounds compared to traditional dressings. Their antimicrobial mechanisms primarily rely on electrical stimulation, zinc ion release, and the generation of reactive oxygen species. These mechanisms can disrupt bacterial cell membranes or interfere with their metabolism, thereby effectively inhibiting bacterial growth. In contrast, conventional cotton dressings, due to their hydrophilic nature, tend to provide a favorable environment for pathogenic bacteria and lack inherent antibacterial activity. Experiments have shown that they fail to form a noticeable zone of inhibition and exhibit almost no ability to suppress or kill bacteria (Wang S. et al., 2024).

Although electrical stimulation offers numerous advantages in promoting wound healing, such as accelerating tissue repair and reducing infection risks, its application still faces certain limitations, particularly regarding cost. Compared to traditional dressings (e.g., gauze, hydrocolloid dressings, etc.), the procurement and maintenance expenses of electrical stimulation devices and related consumables are considerably higher. Additionally, the treatment process often requires professional operation, further increasing medical costs. Therefore, in routine wound care or resource-limited healthcare settings, economical and convenient traditional dressings remain the more common choice. Moving forward, to facilitate the widespread adoption of electrical stimulation technology, the key lies in reducing costs through technological optimization and large-scale production or developing more affordable portable devices to enhance clinical accessibility (Nicksic et al., 2022).
